# Clinical predictors of suicidal ideation, suicide attempts and suicide death in depressive disorder: a systematic review and meta-analysis

**DOI:** 10.1007/s00406-023-01716-5

**Published:** 2023-11-28

**Authors:** Pau Riera-Serra, Guillem Navarra-Ventura, Adoración Castro, Margalida Gili, Angie Salazar-Cedillo, Ignacio Ricci-Cabello, Lorenzo Roldán-Espínola, Victoria Coronado-Simsic, Mauro García-Toro, Rocío Gómez-Juanes, Miquel Roca

**Affiliations:** 1 Research Institute of Health Sciences (IUNICS), University of the Balearic Islands (UIB), Palma, Balearic Islands Spain; 2 Health Research Institute of the Balearic Islands (IdISBa), Son Espases University Hospital, Palma, Balearic Islands Spain; 3 Balearic Islands Health Services (IB-SALUT), Primary Care Research Unit of Mallorca, Palma, Balearic Islands Spain; 4 CIBER Epidemiology and Public Health (CIBERESP), Madrid, Spain; 5 Department of Medicine, University of the Balearic Islands, Palma, Balearic Islands Spain

**Keywords:** Suicide, Depression, Meta-analysis, Prevention, Predictors, Clinical

## Abstract

Patients with depressive disorders are especially prone to suicide risk. Among the clinical predictors of suicidality, those specifically related to depressive disorders have not been accurately detailed. Our aim was to conduct a systematic review and meta-analysis of studies reporting longitudinal predictors of suicidal ideation, suicide attempts and suicide death within depression, including diagnostic subtypes, symptoms, clinical course, and assessment scales. A systematic search of the literature between 2001 and 2022 identified 4422 references, among which 19 studies providing 45 different predictors of suicidality met the inclusion criteria. Random effects meta-analyses were performed for 22 predictors, three for suicidal ideation, eleven for suicide attempts and eight for suicide death. Heterogeneity and publication bias were inspected through *I*^*2*^ tests and Egger’s tests respectively. Meta-analysis results showed that severity of hopelessness predicted suicidal ideation and suicide attempts. History of suicide attempts, suicidal ideation, severe depression, and psychotic symptoms predicted subsequent suicide attempts and suicide death. Time to full remission and sleep disturbances were also found as relevant predictors of future suicide behaviours. This review specifies which predictors of suicidality within the clinical features of depression will help clinicians and policy makers to better prevent suicide risk in patients with depressive disorders. Further longitudinal studies are needed to reliably assess the predictive ability of our results and to analyse other possible clinical predictors to prevent suicidality, especially with regard to suicidal ideation.

**Supplementary Information:**

The online version contains supplementary material available at 10.1007/s00406-023-01716-5.

## Introduction

Every year more than 700,000 people die by suicide worldwide, accounting for 1.4% of all-cause deaths [1, 2]. Research on suicide predictors can help clinicians better detect high-risk individuals and enhance the effectiveness of suicide prevention programs [3]. Patients with depressive disorders are especially vulnerable to suicide [4] and suicidal behaviour [5–7]. Suicide attempts are found to be fivefold more common in patients with depression than in the general population [8]. Recent systematic reviews have estimated a pooled lifetime prevalence of suicide attempts in patients with major depressive disorder (MDD) to be around 31% [9], and a suicidal ideation prevalence of nearly 38% [10].

Evidence suggests that suicidal ideation, suicide attempts and suicide death may have distinct predictors [11], so further studies were developed on the specific protective and risk factors for these three suicidal outcomes. To date, a first meta-analysis by Hawton et al. [12] featuring 19 studies of patients with depressive disorders, reported exclusively predictors of suicide deaths. A more comprehensive approach was provided by the meta-analysis of Li et al. [13], which examined predictors of suicidal ideation, suicide attempts and suicide death. This study identified twenty-nine predictors (ten for suicidal ideation, thirteen for suicide attempts, and six for suicide death) from 24 studies covering 954,822 participants with MDD. Despite its obvious strengths over the first meta-analysis, this second meta-analysis broadly covers sociodemographic, environmental, behavioural, and medical history factors of suicidality, and does not break down the clinical features of depression into its more specific characteristics (e.g., guilt, pessimism, loss of concentration, severity of hopelessness, melancholic traits, time in depression, time to remission).

Previous longitudinal studies have linked these clinical predictors to an increased suicidality in patients with depressive disorders, both for suicidal ideation [14–17] and for suicide attempts [18–24] and suicide death [25–31]. Current knowledge of these depression-related clinical predictors has only been systematically reviewed for the suicide death outcome [12] or in the general population [32]. Therefore, our aim is to conduct a systematic review and meta-analysis of studies reporting at least one longitudinal clinical predictor of suicidality among adults with depressive disorders, breaking down the broad concept of depression predictors into its most specific features (i.e., diagnosis subtype, clinical symptoms, clinical course, and clinical assessment scales). More specifically, we aim to assess the impact of these depression-related clinical predictors on three suicidal outcomes: suicidal ideation, suicide attempts, and suicide death.

## Methods

The present study is reported following the Preferred Reporting Items for Systematic and Meta-analysis (PRISMA) guidelines [33]. We previously registered the original research protocol at the PROSPERO International Prospective Register of Systematic Reviews (registration number CDR42022319840) on April 21, 2022.

### Search strategy

Published studies were systematically searched in PubMed (Medline), EMBASE, PsycINFO and Cochrane Library electronic databases. We searched for grey literature in OpenGrey Website, Open Access Theses and Dissertations (OATD) and Web of Science (Science Citation Index) to not overlook relevant yet unpublished results. All literature registered from January 1, 2001, to October 3, 2022, was searched. We developed Boolean combinations of key terms and MeSH terms (e.g. “depressive disorder”, “suicide”, “prospective study”) inspired by the search strategies of prior systematic reviews [6, 12, 32, 34]. All the search terms, keywords and results can be found in the Supplementary Material (Table S1–S8). The reference lists of relevant studies and reviews were scrutinized for papers not yet identified.

### Eligibility criteria

Inclusion criteria for the studies were as follows: (a) study population aged 18 years or older; (b) diagnosis of depressive disorder at the time of study entry according to internationally validated criteria (i.e., International Classification of Diseases, ICD; Diagnostic and Statistical Manual of Mental Disorders, DSM); (c) published in English; (d) discrete suicidality measures (suicidal ideation, suicide attempt or suicide death); (e) predictors of suicidal ideation, suicide attempts or suicide death related to depression or suicidality itself; (f) quantitative data (e.g., test result) comparing depressed patients with suicidality (cases) and depressed patients without suicidality (controls); and (g) cohort design or case–control design (the latter was only valid for studies with suicide death outcomes).

Only studies whose designs ensured that predictors preceded the outcome of interest were selected. This is mandatory for considering selected predictors as risk or protective factors, and not as correlates [35]. In case–control studies in which the cases are subjects with suicidal ideation or suicide attempts, predictors can often be assessed at the same time as the occurrence of these outcomes. Thus, only longitudinal cohort studies, or case–control studies whose outcome of interest was suicide death, were eligible. Importantly, cohort studies were included in this review regardless of whether these reported on predictors of short- or long-term suicidality.

Studies were excluded when populations had: (a) intellectual disability; (b) neurological disorder (e.g., brain damage, dementia, epilepsy) or neurodevelopmental disorders (e.g., autism); and (c) serious mental illness other than depression (e.g., schizophrenia spectrum disorders, bipolar disorder) according to DSM or ICD criteria. However, depressed samples with less than 20% of patients diagnosed with bipolar disorder were accepted since roughly this proportion of subjects with a diagnosis of MDD may eventually develop symptoms of mania or hypomania [36]. We further excluded (d) cross-sectional studies and clinical trials; (e) study protocols, editorials, conference abstracts with no full-text available and letters to the editor; and (f) studies with too small samples of patients with depressive disorders (*N* ≤ 20).

### Screening process

References were extracted from the databases and duplicates were automatically and manually removed through EndNote reference manager. The Rayyan systematic review platform [37] was used to conduct the screening process. Titles and abstracts were screened by five groups of two independent peer reviewers and conflicts were resolved with support from a third reviewer (AC, ASC, LRE, MG, MGT, MR, PRS, RGJ, VCS). Full texts of the articles selected in the previous screening were reviewed using the same strategy and the reason for exclusion was specified. Study authors were contacted when more detailed data was needed, or when full text was not available.

### Data extraction

Data from the included studies was extracted by one author and all data was cross-checked by a second peer reviewer for accuracy and completeness (AC, ASC, LRE, PRS, VCS). Any discrepancy between authors was resolved through consensus among reviewers. Details from publication (e.g., authors, year, country), study design (e.g., follow-up length), sample characteristics (e.g., sample age, number of participants with depression), suicidal measures (e.g., inventories) and predictors (e.g., description; relevant statistics) were obtained and codified for each independent study sample.

We established the following terminological distinction to refer to the collected data on predictors of suicidality. A “prediction case” (*k*) was any quantitative test result (e.g., odds ratios, means difference) on a predictor extracted from the included studies. A “predictor” was any longitudinal factor predicting suicidality within the clinical features of depression (e.g., hopelessness, severity of depression, time in depression). Finally, collected predictors were subsequently classified into broader “predictor categories” (i.e., diagnostic subtype, clinical symptoms, clinical course, and clinical assessment scale). Table S9 provides more details on the classification of the predictors found among the included studies.

Importantly, when a same predictor was reported within a study in several publications or multiple follow-ups, those studies reporting larger sample and/or longest follow-up were preferred. In addition, pre-operationalization of sixteen predictors was performed prior to the meta-analysis. First, analogous predictors (e.g., Beck Depression Inventory, BDI; Hamilton Depression Rating Scale, HDRS) were combined under broader predictors (e.g., severity of depression) for better conciseness and robustness of the meta-analysis results. Second, assessment scales (e.g., Beck Hopelessness Scale, BHS) found as predictors of suicidality were relabelled according to the construct under assessment (e.g., severity of hopelessness) for better intelligibility of the results. Further details are provided in Table S10.

### Risk of bias

The quality of the included studies was assessed by two groups of two independent peer reviewers (AC, ASC, LRE, VCS) using the Newcastle–Ottawa Scale (NOS) adapted for cohort and case–control designs [38]. The discrepancies were resolved through the intervention of the first author of the manuscript (PRS). When the same study reported different suicidality outcomes, the NOS assessment was performed for each outcome. NOS evaluates three different domains using a “star system” score: (a) selection of the study groups; (b) comparability of the groups; and (c) the ascertainment of either the outcome or the exposure for cohorts and case-controls designs respectively. The episodic nature of suicidal behaviours posed a challenge in accurately assessing NOS criterion S4, which evaluates whether the study outcomes were already present at baseline. To address this limitation, we assigned a positive rating to this criterion only for studies in which a minority of participants (≤ 10%) had a history of the examined suicidal outcome at baseline. The total NOS score ranges from 0 to 9, with studies scoring less than 6 being considered low quality.

### Statistical analysis

First, selected characteristics of the included articles were synthesized and tabulated. Meta-analyses of the predictors were conducted with STATA 17.0. *metan* package was used to pool estimates of Odds Ratios (ORs) and Hazard Ratios (HRs), the latter only for cohort studies. Meta-analyses were performed for each suicidal outcome (ideation, attempts, and death). At least two primary studies had to report prediction cases (*k* ≥ 2) on the same suicidal outcome for meta-analytical calculation. When one study reported multiple measures of depression severity scales, BDI scale scores were preferred as being more frequently found, but the analyses were then replicated with the prediction cases from the other scales (i.e., HDRS). When ORs were not reported, these were calculated from contingency tables or from independent group means, standard deviations and sample size [39]. Similarly, confidence intervals and standard errors were calculated from *p* values and *z *scores when these were not provided [40]. Following Ribeiro et al. [32] systematic review, zero-order unadjusted effects of ORs and HRs were preferred over adjusted effects as a purer measure of the effect. Heterogeneity was quantified using *I*^*2*^ tests and it was also inspected visually throughout forest plots. Substantial heterogeneity was considered when *I*^*2*^ was higher than 50% [41]. We conducted random-effects models for the pooled effects using DerSimonian and Laird method and assuming between-study variances. Finally, funnel plots were examined to detect publication bias of predictors, and Egger’s tests were conducted to test asymmetry in the meta-analyses. No subgroup analysis or multivariate meta-analysis was performed due to the relative scarcity of prediction cases per predictor.

## Results

### Study selection

**Fig. 1:**
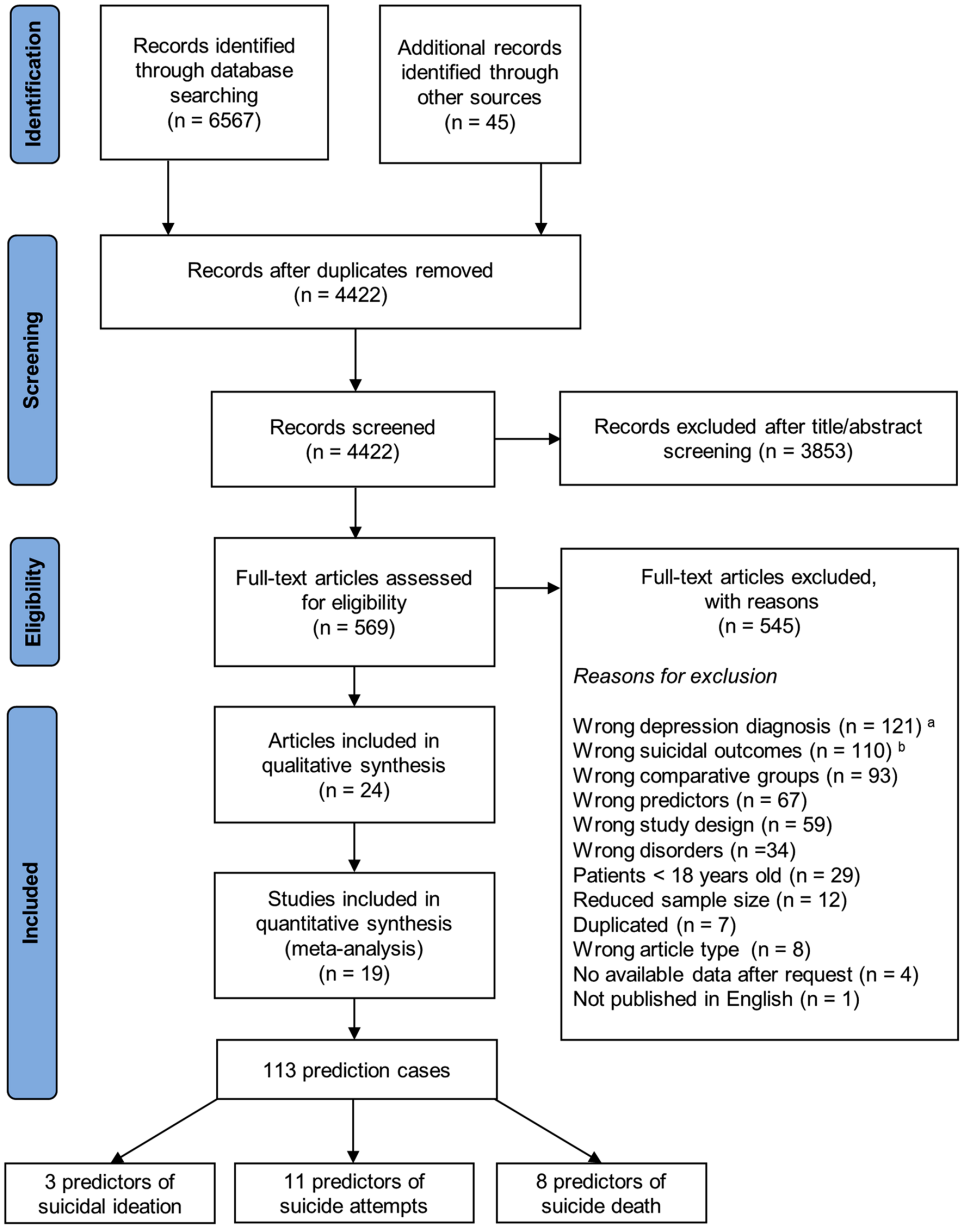
PRISMA flowchart. ^a^ Studies whose participants did not have a diagnosis of depressive disorder according to internationally validated criteria (i.e., DSM, ICD) at the time of the study entry were not included. ^b^ Studies that reported only general outcomes of suicidality (e.g., suicidal risk, suicidal behaviour), a history of suicidal ideation or attempts, or measures of self-injury without suicidal intent were excluded

A total of 4422 unique references were identified throughout the search strategy. Of these, 3853 were excluded after screening of titles and abstracts, and 545 were excluded after full-text review. Flow-chart of the selection process is shown in Fig. 1. As a result, we identified 24 publications [14–23, 25–29, 42–50] reporting results from 19 different studies. One included study [14] owes its sample to the combination of patients recruited from two different studies also covered by this systematic review: the “Vantaa Depression Study” [17, 21, 23] and the “Vantaa Primary Care Depression Study” [22]. Thus, data from the study conducted by Baryshnikov et al. [14] was excluded when this involved a duplication and, therefore, misestimating the aggregated results (e.g., sample, mean age, mean follow-up time), but was deemed as an independent study when it provided additional relevant information (e.g., suicidality predictors).

### Systematic review

Overall, 19 studies reported quantitative data on depression-related suicidality predictors from 340,039 adults with clinically diagnosed depression. Aggregated features of the included studies are displayed in Table 1. Among studies reporting sufficient data (*N* = 16), mean age of participants ranged from 31.4 (SD = 9.0) to 67.8 (SD = 6.1) years old, with an overall weighted mean age of 50.35 (SD_w_ = 3.19) years old. Ten studies (reported in 15 publications) provided data from individuals with MDD [15, 17, 19–21, 23, 26, 28, 29, 43–45, 47, 48, 50], six from mixed depressive disorder samples [14, 16, 18, 22, 25, 27], two from patients with treatment-resistant depressive disorder [42, 49], and one from patients with a diagnosis of severe depression [46]. Thirteen studies (reported in 16 publications) included psychiatric inpatients [15, 17–21, 23, 25–27, 43–47, 50], four studies (reported in five publications) patients from specialized units, university centres or psychiatric outpatients clinics [16, 28, 29, 48, 49], one study patients from primary care centres [22], and another study was a mixed sample collected from inpatients and outpatients [14]. Publication years ranged from 2001 to 2022, with no statistically significant differences found in the number of articles published every 5 years (*χ*^2^ = 8.00; *P* = 0.238). Included studies were conducted in 10 different countries; more than half were conducted in Europe (*N* = 11), and predominantly in the Nordic countries (*N* = 8). The mean follow-up of the cohort studies was 7.94 years, ranging from 6 weeks to 24 years. Table 2 provides a selection of characteristics of each study. The mean NOS score of the included studies was 6.76 (SD = 1.17), and the majority of the studies (*N* = 14) had an NOS score above 6, which is considered to be “high quality”. Further details of the NOS quality assessment for each study can be found in Table S11.

**Table 1:** Selected characteristics of the studies included in the systematic review (*N* = 19)

	*N*	%
Year of study publication^a^ (*N* = 24)		
2001–2005	5	20.83
2006–2010	5	20.83
2011–2015	6	25.00
2016–2022	8	33.33
Continent^b^ (*N* = 18)		
Europe	11	61.11
North America	5	27.78
Asia	2	11.11
Study design^b^ (*N* = 18)		
Cohorts	15	83.33
Case-controls	3	16.67
Study length^c^ (*N* = 16)		
≤ 1 year	6	37.50
≤ 5 years	4	25.00
≤ 10 years	3	18.75
> 10 years	3	18.75
Participant source (*N* = 19)		
Inpatient	5	26.32
Outpatient	5	26.32
Inpatients/outpatients	6	31.58
Others	3	15.79
Sample mean age^a^ (*N* = 24)		
18–40 years old	5	20.83
40–60 years old	16	66.67
> 60 years old	1	4.17
≥ 18 (undefined)	2	8.33
Depressed sample (*N*)^b,d^ (*N* = 18)	340,039	n/a
Diagnostic criteria^b^ (*N* = 18)		
DSM	12	66.67
ICD	6	33.33
Suicide outcomes^e^ (*N* = 22)		
Suicidal ideation	5	22.73
Suicide attempt	8	36.36
Suicide death	9	27.27
ROB (NOS)^f^ (*N* = 22)		
< 5	0	0.00
5	5	22.73
6	3	13.64
7	9	40.91
8	4	18.18
9	1	4.55

*n/a* Not applicable, *DSM* Diagnostic and Statistical Manual of Mental Disorders, *ICD* International Classification of Diseases, *ROB* Risk of Bias; *NOS* Newcastle–Ottawa Scale

^a^Data reported for each publication included in the systematic review

^b^Baryshnikov et al. (2020) data was excluded for this analysis

^c^Maximum follow-up of the cohort articles included in each study

^d^Maximum number of participants of the articles included in each study

^e^Two studies reported more than one suicidal outcome

^f^Risk of bias was assessed for each suicidal outcome

**Table 2:** Characteristics of studies included in the systematic revie﻿w (*N* = 19)

No.	Authors (publication year)	Country	Study design	Follow-up period^a^	Sample setting	Age (years)^b^	Depressive sample (N)	Diagnostic criteria	Suicide outcome	Suicide outcomes source	Suicidal cases	Non-suicidal controls	Depression-related predictors^c^
1	Aaltonen et al. (2019)	Finland	Cohort	24 years	Inpatients	44.8 (17.5)	56,826	ICD-9 ICD-10	Death	National register	2,587	54,239	Severe depression; psychotic features
2	Baryshnikov et al. (2020)	Finland	Cohort	5 years	Inpatients/outpatients	41.6^d^ (12.3)	406	DSM-IV	Ideation	BSSI	n/a^e^	n/a^e^	Severity of depression (MADRS); severity of hopelessness (BHS)
3	Chan et al. (2014)	Malaysia	Cohort	1 year	Inpatients	43.8 (12.1)	75	DSM-IV	Attempt	Medical records and clinical interviews	12	54	History of suicide attempt; severity of suicidal ideation (BSSI); severity of depression (BDI); psychotic features; melancholic features; family history of suicidal behaviour
4.1	Courtet et al. (2014)	France	Cohort	6 weeks	Outpatients	47.3 (13.9)	4,357	DSM-IV	Ideation	MADRS item 10	81	838	History of suicide attempt; severity of depression (MADRS); severity of hopelessness (BHS): number of past MDE; age at first diagnosis of MDD; time in depression
4.1	Courtet et al. (2014)	France	Cohort	6 weeks	Outpatients	47.3 (13.9)	4,357	DSM-IV	Attempt	clinical interviews	75	4,282	History of suicide attempt; increase of suicidal ideation; severity of suicidal ideation (BSSI); severity of depression (BDI); severity of hopelessness (BHS): number of past MDE; age at first diagnosis of MDD; time in depression
5	Galfalvy et al. (2008); Grunebaum et al. (2004)	United States	Cohort	2 years	Inpatients/outpatients	38.0 (12) 40.1 (13.1)	304 377	DSM-III-R DSM-IV	Attempt	clinical interviews (CSHF)	52 44	184 220	History of suicide attempt; severity of suicidal ideation (BSSI); severity of depression (BDI); severity of hopelessness (BHS); melancholic featuress
6	Gladstone et al. (2001)	Australia	Cohort	10 years	Inpatients/outpatients	≥ 18	813	DSM-III DSM-III-R DSM-IV	Death	Medical records and death register	31	782	Hopelessness; pessimism
7	Gronemann et al. (2021); Kessing (2004)	Denmark	Cohort	15 years 6 years	Inpatients/outpatients	52.4^d^ (21.1) ≥ 18	195,539 7,199	ICD-10	Death	National death register	1,432 81	194,107 7,118	Severe depression
8.1	Sokero et al. (2006)	Finland	Cohort	26 weeks	Inpatients/outpatients	38.9 (10.1)	70	DSM-IV	Ideation	BSSI	15	47	Severity of depression (BDI); severity of hopelessness (BHS); severity of suicidal ideation (BSSI)
8.2	Holma et al. (2010);	Finland	Cohort	5 years	Inpatient / outpatient	39.6 (11.1)	249	DSM-IV	Attempt	Clinical interview and medical records	36	213	History of suicide attempt; number of past suicide attempts; suicidal ideation; severity of suicidal ideation (BSSI); severity of depression (BDI; HDRS); severity of hopelessness (BHS); psychotic features; melancholic features; atypical features; dysthymia; current MDE; age at diagnosis of MDD; time to full remission of MDE

No.	Authors (publication year)	Country	Study design	Follow-up period^a^	Sample setting	Age (years)^b^	Depressive sample (*N*)	Diagnostic criteria	Suicide outcome	Suicide outcomes inventory	Suicidal cases	Non-suicidal controls	Depression-related predictors^c^
8.2	Sokero et al. (2005)	Finland	Cohort	18 months	Inpatients/outpatients	41 (11.1)	269	DSM-IV	Attempt	Clinical interview and medical records	16	182	Severity of depression (BDI; HDRS); severity of hopelessness (BHS); number of past MDE; time in depression; time to full remission of MDE
9	Kim et al. (2012)	United States	case–control	n/a	Veterans	56 (13.4)	636	ICD	Death	Death register	324	312	History of suicide attempt; depression symptomatology; suicidal ideation; suicidal plan; psychotic features; sleep complaints; time since depression diagnosis
10	Leadholm et al. (2014)	Denmark	Cohort	16 years	Outpatients	51.3^d^ (18.4)	34,671	ICD-10	Death	Medical records	755	33,916	History of suicide attempt; psychotic depression; psychotic features; age at diagnosis of depression
11	Manning et al. (2021)	United States	Cohort	10 years	Outpatients	67.8 (6.1)	112	DSM-IV	Ideation	MADRS item 10	n/a^e^	n/a^e^	History of suicide attempt; severity of depression (MADRS)
12	McGirr et al. (2007); McGirr et al. (2008)	Canada	case–control	n/a	Outpatients / community	42.5^d^ (12.5) 40.9^d^ (12.4)	237 297	DSM-IV	Death	Death register	156 154	81 143	suicidal ideation / thoughts of death; severe depression; psychotic features; insomnia; depressed mood; hypersomnia; depressed mood; psychomotor disturbance; worthlessness or guilt; fatigue, anhedonia; appetite gain; decreased appetite; loss of concentration; history of depressionration
13	Nikel et al. (2006)	Germany Austria	Cohort	1 year	Community/ inpatients/outpatients	33.4 (5.1)	425	DSM-IV	Attempt	Clinical interview	126	299	History of suicide attempt; hopelessness
14	Nobile et al. (2022)	France	Cohort	1 year	Inpatients	40.3 (14.4)	898	DSM-V	Attempt	Medical records	72	446	Suicidal ideation; severity of depression (BDI; IDS)
15.1	Qiu et al. (2017)	United States	Cohort	10 years	Outpatients	31.4^d^ (9.0)	142	DSM-III-R	Ideation	LIFE interview	85	17	Severity of hopelessness (BHS)
15.2	Qiu et al. (2017)	United States	Cohort	10 years	Outpatients	31.4^d^ (9.0)	142	DSM-III-R	Attempt	LIFE interview	85	11	Severity of hopelessness (BHS)
16	Reutfors et al. (2021)	Sweden	case–control	n/a	Inpatients/outpatients	18–70	15,631	ICD-10	Death	Death register	178	534	History of suicide attempt; history of depression
17	Riihimäki et al. (2014)	Finland	Cohort	5 years	Outpatients	45.3 (13.6)	134	DSM-IV	Attempt	Medical records and clinical interview	14	120	History of suicide attempt; suicidal ideation; severity of suicidal ideation (BSSI); severity of depression (BDI; HDRS); severity of hopelessness (BHS); time in depression; time in full remission of MDE; number of past MDE
18	Rönnqvist et al. (2021)	Sweden	Cohort	1 year	Inpatients	47.6^d^ (15.1)	28,557	ICD-10	Death	Medical records	356	28,201	Severe depression; psychotic features
19	Schneider et al. (2001)	Germany	Cohort	5 years	Inpatients	44.2 (14.7)	280	DSM-III-R	Death	Relatives reports and medical records	16	262	Severe depression; psychotic features; melancholic subtype; insomnia; agitation; sexual interest; weight loss; guilt; retardation; age at diagnosis of MDD

*BDI* Beck Depression Inventory, *BHS* Beck Hopelessness Scale, *BSSI* Beck Scale for Suicide Ideation, *CSHF* Columbia Suicide History Form, *DSM* Diagnostic and Statistical Manual of mental disorders, *HADS* Hospital Anxiety and Depression Scale, *HDRS* Hamilton Depression Rating Scale, *ICD* International Classification of Diseases, *IDS* Inventory of Depressive Symptomatology, *LIFE* Longitudinal Interval Follow-up Evaluation, *MADRS* Montgomery-Åsberg Depression Rating Scale, *MDD* major depression disorder, *MDE* major depressive episode, *NOS* Newcastle–Ottawa scale, *n/a* Not applicable, *SD* standard deviation

^a^Maximum follow-up length of the study

^b^Mean age and SD (in parenthesis) of the study participants are reported

^c^Predictors are reported as labelled in subsequent meta-analyses Only predictors with enough quantitative data to allow comparison of groups were reported

^d^Mean age and SD was estimated from different study subgroups data

^e^The number of cases and controls are susceptible to variation due to type of analysis conducted (i.e., Generalized Linear Mixed Models)

Among the included studies, we collected a total of 45 different predictors of suicidality. The most frequent predictors reported were a personal history of suicide attempt (*k* = 11*),* the BDI (*k* = 8) and the Beck Hopelessness Scale (*k* = 8). According to our pre-established classification of predictor categories, we found 5 predictors from the diagnostic subtype category (*k* = 19), 23 predictors from the clinical symptoms category (*k* = 31), 10 predictors from the clinical course category (*k* = 35), and 7 predictors from the clinical assessment scales category (*k* = 28). Further details are provided in Table S9.

### Meta-analyses

The 45 predictors of suicidality found among the included studies were supported by a total of 113 prediction cases (82 extracted as OR, 13 extracted as HR, and 18 as both). After the pre-operationalization process, 37 of the 45 collected predictors were retained. However, since most predictors were not supported by more than one study, only 22 predictors were ultimately meta-analysed. In total, three predictors of suicidal ideation (*k* = 8), 11 predictors of suicide attempt (*k* = 37), and eight predictors of suicide death (*k* = 29) were meta-analysed. The meta-analyses results on ORs are summarized in Table 3. The meta-analyses results on HRs are presented in Table S12.

**Table 3:** Meta-analysis of odds ratios (ORs) for depression-related predictors of suicidal ideation, suicide attempts and suicide death

Outcome	Predictors	Study (year)	OR (95% CI)	Weight	wOR (95% CI)	Heterogeneity
Suicidal ideation	History of suicide attempt	Courtet et al. (2014)	1.19 (0.46–3.08)	51.20%	1.75 (0.81–3.77) *P* = 0.156	*I*^2^ = 21.23% *χ*^*2*^ = 1.27; *P* = 0.260
		Manning et al. (2021)	2.61 (0.98–6.95)	48.80%		
	Severity of depression	Baryshnikov et al. (2020)	4.43 (3.08–6.71)	31.97%	1.68 (0.82–3.53) *P* = 0.156	*I*^2^ = 96.00% *χ*^*2*^ = 50.06; *P* < 0.001
		Courtet et al. (2014)	1.09 (0.79–1.50)	32.93%		
		Manning et al. (2021)	1.07 (1.02–1.13)	35.10%		
	Severity of hopelessness	Baryshnikov et al. (2020)	2.80 (2.22–3.55)	45.97%	2.53 (1.43–4.47) *P* = 0.001	*I*^2^ = 70.08% *χ*^*2*^ = 6.69; *P* = 0.035
		Courtet et al. (2014)	1.43 (0.82–2.51)	33.68%		
		Qiu et al. (2017)	5.17 (1.96–13.65)	20.35%		
Suicide attempt	History of suicide attempts	Chan et al. (2014)	4.71 (1.14–19.44)	10.20%	3.72 (2.15–6.42) *P* < 0.001	*I*^2^ = 71.78% *χ*^*2*^ = 14.18; *P* = 0.007
		Courtet et al. (2014)	5.75 (3.61–9.17)	26.35%		
		Holma et al. (2010)	3.74 (1.80–7.78)	20.56%		
		Nikel et al. (2006)	2.13 (1.15–2.04)	29.92%		
		Riihimäki et al. (2014)	4.54 (1.40–14.73)	12.98%		
	Suicidal ideation	Holma et al. (2010)	2.19 (0.95–5.04)	35.32%	2.44 (1.48–3.99) *P* < 0.001	*I*^2^ = 0% *χ*^*2*^ = 0.10; *P* = 0.757
		Nobile et al. (2022)	2.58 (1.39–4.76)	64.68%		
	Severity of suicidal ideation	Chan et al. (2014)	5.48 (1.7017.70)	29.05%	3.09 (0.79–12.11) *P* = 0.105	*I*^2^ = 93.8% *χ*^*2*^ = 32.10; *P* < 0.001
		Holma et al. (2010)	5.90 (3.05–11.41)	34.03%		
		Riihimäki et al. (2014)	1.08 (0.58–2.01)	36.92%		
	Severity of depression	Chan et al. (2014)	2.13 (0.68–6.68)	6.11%	2.16 (1.63–2.86) *P* < 0.001	*I*^2^ = 0% *χ*^*2*^ = 1.23; *P* = 0.745
		Courtet et al. (2014)	1.95 (1.38–2.75)	67.06%		
		Holma et al. (2010)	2.67 (1.40 –5.11)	19.07%		
		Riihimäki et al. (2014)	3.08 (1.12–8.48)	7.76%		
	Severity of hopelessness	Courtet et al. (2014)	1.08 (0.58–2.01)	32.41%	2.08 (1.19–3.66) *P* = 0.011	*I*^2^ = 47.9% *χ*^*2*^ = 5.76; *P* = 0.124
		Holma et al. (2010)	2.65 (1.37–4.99)	31.40%		
		Qiu et al. (2017)	2.75 (0.87–8.67)	16.62%		
		Riihimäki et al. (2014)	3.39 (1.23–9.37)	19.57%		
	Psychotic features	Chan et al. (2014)	2.86 (0.80–10.33)	29.27%	3.17 (1.59–6.33) *P* < 0.001	*I*^2^ = 0% *χ*^*2*^ = 0.04; *P* = 0.851
		Holma et al. (2010)	3.31 (1.45–7.54)	70.73%		
	Melancholic features	Chan et al. (2014)	0.34 (0.09–1.28)	38.36%	0.70 (0.23–2.15) *P* = 0.535	*I*^2^ = 56.87 *χ*^*2*^ = 2.32; *P* = 0.128
		Holma et al. (2010)	1.10 (0.53–2.28)	61.64%		
	Time in depression	Courtet et al. (2014)	1.10 (0.73–1.66)	38.09%	2.74 (0.87–8.59) *P* = 0.084	*I*^2^ = 84.52 *χ*^*2*^ = 12.92*; P* = 0.002
		Riihimäki et al. (2014)	4.94 (1.78–13.7)	30.36%		
		Sokero et al. (2005)	4.70 (1.84–12.02)	31.55%		

Outcome	Predictors	Study (year)	OR (95% CI)	Weight	wOR (95% CI)	Heterogeneity
Suicide attempt	Age at diagnosis of MDD	Courtet et al. (2014)	0.87 (0.73–1.03)	93.28%	0.86 (0.73–1.01) *P* = 0.068	*I*^2^ = 0% *χ*^*2*^ = 0.47; *P* = 0.493
		Holma et al. (2010)	0.69 (0.36–1.31)	6.72%		
	Time to full remission of MDE	Riihimäki et al. (2014)	3.84 (1.39–10.61)	46.00%	4.16 (2.09–8.30) *P* < 0.001	*I*^2^ = 0% *χ*^*2*^ = 0.05; *P* = 0.830
		Sokero et al. (2005)	4.46 (1.75–11.42)	54.00%		
	Number of past MDE	Holma et al. (2010)	1.14 (0.60–2.15)	71.07%	1.15 (0.67–1.98) *P* = 0.604	*I*^2^ = 0% *χ*^*2*^ = 0.01; *P* = 0.926
		Riihimäki et al. (2014)	1.20 (0.44–3.28)	28.93%		
Suicide death	History of suicide attempt	Kim et al. (2012)	17.91 (5.52–58.16)	8.05%	4.85 (3.32–7.08) *P* < 0.001	*I*^2^ = 81.49% *χ*^*2*^ = 16.21; *P* = 0.001
		Leadholm et al. (2014)	4.90 (4.19–5.73)	34.20%		
		Reutfors et al. (2021)	4.45 (2.52–7.86)	26.56%		
		Ronnqvist et al. (2021)	3.07 (2.38–3.96)	31.20%		
	Suicidal ideation or thoughts of death	Kim et al. (2012)	3.34 (2.17–5.13)	55.28%	6.05 (1.66–22.01) *P* = 0.006	*I*^2^ = 83.97% *χ*^*2*^ = 6.24; *P* = 0.012
		McGirr et al. (2007)	12.59 (4.88–32.47)	44.72%		
	Severe depression	Kessing (2004)	2.34 (1.50–3.64)	28.15%	1.74 (1.14–2.65) *P* = 0.010	*I*^2^ = 66.85% *χ*^*2*^ = 9.05;* P* = 0.029
		McGirr et al. (2007)	1.27 (0.67–2.40)	21.15%		
		Ronnqvist et al. (2021)	1.29 (1.04–1.60)	13.87%		
		Schneider et al. (2001)	3.39 (1.35–8.53)	36.84%		
	Psychotic features	Kim et al. (2012)	4.00 (2.12–7.54)	19.04%	1.57 (1.06–2.33) *P* = 0.025	*I*^2^ = 70.4% *χ*^*2*^ = 13.52;* P* = 0.009
		Leadholm et al. (2014)	1.35 (1.10–1.64)	34.33%		
		McGirr et al. (2007)	2.07 (0.56–7.75)	7.21%		
		Ronnqvist et al. (2021)	1.15 (0.88–1.51)	31.93%		
		Schneider et al. (2001)	0.83 (0.23–3.01)	7.48%		
	Guilt	McGirr et al. (2007)	2.40 (1.344.30)	62.78%	1.84 (0.94–3.61) *P* = 0.076	*I*^2^ = 39.34% *χ*^*2*^ = 1.65; *P* = 0.200
		Schneider et al. (2001)	1.18 (0.47–2.91)	37.22%		
	Sleep disturbances	Kim et al. (2012)	1.35 (0.97–1.88)	61.61%	1.64 (1.13–2.38) *P* = 0.009	*I*^2^ = 23.13% *χ*^*2*^ = 2.60; *P* = 0.272
		McGirr et al. (2007)	2.37 (1.21–4.66)	24.04%		
		Schneider et al. (2001)	2.08 (0.83–5.21)	14.35%		
	Previous history of depression	McGirr et al. (2008)	6.02 (3.64–9.97)	50.20%	2.62 (0.51–13.49) *P* = 0.250	*I*^2^ = 94.89% *χ*^*2*^ = 19.56; *P* < 0.001
		Reutfors et al. (2021)	1.13 (0.66–1.96)	49.80%		
	Age at diagnosis of MDD	Leadholm et al. (2014)	1.25 (1.09–1.42)	98.00%	1.24 (1.09–1.41) *P* = 0.001	*I*^2^ = 0% *χ*^*2*^ = 0.25; *P* = 0.620
		Schneider et al. (2001)	0.99 (0.40–2.47)	2.00%		

*OR* odds ratio, *wOR* weighted odds ratio, *MDD* major depressive disorder, *MDE* major depressive episode

All studies provided some predictor that was ultimately meta-analysed except the study of Gladstone et al.  [26], which provided two predictors that were not reported in any other study. The mean number of prediction cases comprising the meta-analysed predictors was relatively low ($\overline{X }$ = 2.87; SD = 1.04), with a maximum number of five studies reporting for a same predictor. Since it is only recommended to analyse publication bias (through Egger's test and funnel plots) in those predictors supported by 10 or more studies [41], results from publication bias tests have been omitted.

### Predictors of suicidal ideation

Regarding suicidal ideation as an outcome, severity of hopelessness was the only significant predictor, with an overall estimated effect of OR_w_ = 2.53 (95% CI 1.43–4.47). Although the *I*^*2*^ statistic for assessing heterogeneity between prediction cases was substantial (*I*^*2*^ = 70.08%), the forest plots of these results showed the same direction of the effect (i.e., risk factor) (Fig. S1). Severity of depression and a previous history of suicide attempts were not found to be relevant predictors of suicidal ideation and heterogeneity among the severity of depression prediction cases was prominent (*I*^*2*^ = 96.00%) (Fig. S1).

### Predictors of suicide attempts

Time to full remission of Major Depressive Episode (MDE) was found to be the most robust predictor of subsequent suicide attempts (OR_w_ = 4.16; 95% CI 2.09–8.30). Five studies provided data for history of suicide attempts predictor, which emerged to be the second greatest predictor of future suicide attempts, with a pooled estimated effect of OR_w_ = 3.72 (95% CI 2.15–6.42). Psychotic features (OR_w_ = 3.17; 95% CI 1.59–6.33), previous suicidal ideation (OR_w_ = 2.44; 95% CI 1.48–3.99), severity of depression (OR_w_ = 2.16; 95% CI 1.63–2.86) and severity of hopelessness (OR_w_ = 2.08; 95% CI 1.19–3.66) were also identified as significant predictors of subsequent suicidal attempts. When meta-analyses were conducted using HDRS scores instead of BDI scores, depression severity remained a significant predictor of suicide attempts (OR_w_ = 1.93; 95% CI 1.46–2.56). Heterogeneity among studies was substantial (*I*^*2*^ > 50%) for history of suicide attempts, severity of suicidal ideation, melancholic features, and time in depression predictors. However, forests plots showed that the prediction cases of all predictors were in the same direction of the effect (i.e., risk factor), except for the melancholic features predictor (Fig. S2).

### Predictors of suicidal death

Suicidal ideation or thoughts of death predicted future suicide death, resulting in a pooled estimated effect of OR_w_ = 6.05 (95% CI 1.66–22.01). Next, history of suicide attempt was the most robust predictor (OR_w_ = 4.85; 95% CI 3.32–7.08), followed by a diagnosis of severe depression (OR_w_ = 1.74; 95% CI 1.14–2.65), sleep disturbances (OR_w_ = 1.64; 95% CI 1.13–2.38), psychotic features (OR_w_ = 1.57; 95% CI 1.06–2.33), and older age at diagnosis of MDE (OR_w_ = 1.24; 95% CI 1.09–1.41). Heterogeneity was substantial except for sleep disturbances, guilt, and age at diagnosis of MDE predictors. However, forest plots inspection showed that the prediction cases of sleep disturbances and guilt predictors were in the same direction of the effect (i.e., risk factor) (Figure S3).

## Discussion

### Summary of main findings

Identifying the most specific predictors of suicidality within the clinical features of depression is a key aspect for the development of more sophisticated strategies for suicide prevention. In this review we systematically integrated and meta-analysed findings from current studies reporting clinical predictors of suicidality in adults with depressive disorders. Our systematic review identified 19 studies yielding a total of 45 different depression-related predictors of suicidality. Of these, 22 predictors were ultimately meta-analysed, three for suicidal ideation, eleven for suicide attempts and eight for suicide death. Studies providing quantitative data on clinical predictors of suicidal attempts and suicide death are much more frequent than those reporting for suicidal ideation. A similar disparity was observed among predictor categories, with more studies providing prediction cases linked to depressive clinical symptoms, clinical course, or clinical assessment scales than diagnostic subtype predictors.

Having a history of suicide attempts, previous suicidal ideation, more severe depression and hopelessness, psychotic symptoms, sleep disturbances, longer time to full-remission and older age at depression diagnosis were found to be relevant predictors of subsequent suicidality in adults with depressive disorders. However, few if any of these predictors were able to reliably predict the targeted suicidality outcomes. The predictive capability of the predictors found in this review is consistent with the results pooled in the meta-analysis by Franklin et al.  [51], which highlighted the absence of robust predictors of suicidality. In addition, the small number of potentially analysable prediction cases, as well as the heterogeneity among them, affect the reliability of our results. Despite substantial heterogeneity for most of the significant predictors of suicidality, the forest plots indicated that the prediction cases were not highly divergent in terms of the direction of the effect, with most suggesting a risk factor for suicidality. All this suggests that the main problems in this field are more the scarcity of studies and the robustness of clinical predictors than the heterogeneity of results among studies.

Importantly, the heterogeneity in follow-ups among the included cohort studies (e.g., 6 weeks vs. 24 years) does affect the interpretation of our results. In particular, the clinical predictors reported in this review should be interpreted as both short- and long-term suicidality predictors. Therefore, we cannot ascertain whether the predicted risk of suicidality in cohort studies with prolonged follow-up ultimately occurred in the context of a depressive episode. However, since clinical features manifested in a first depressive episode are likely to be present in subsequent episodes [52], the clinical predictors of suicidality reported in this review may be relevant to both current and future depressive episodes.

### Clinical predictors of suicidality

Hopelessness emerged as a predictor for both subsequent suicidal ideation and suicide attempts, more robustly predicting suicidal ideation than future suicide attempts. Our results are in line with those reported by the meta-analysis of longitudinal studies conducted by Ribeiro et al. [32]. However, other non-included studies with populations with depression reported contradictory evidences regarding this association [53, 54]. The recent meta-analysis by Li et al.  [13] found no significant relationship between hopelessness and suicidal behaviours. The limited and inconsistent results found in these two systematic reviews call for further longitudinal studies on this predictor. Recent findings suggest that hopelessness could amplify the positive relationship between psychiatric symptoms and suicidal ideation [55]. Moreover, Baryshnikov et al. [14] indicate that the strength of hopelessness in predicting suicidal ideation was attenuated when the models were controlled for psychiatric symptoms, such as depression or anxiety. These findings open new study horizons for better understanding the role of hopelessness in suicide risk and suggest that the effect of psychiatric symptoms should be controlled when analysing this association.

Previous suicidal behaviours, either suicidal ideation or suicide attempts, were found to be statistically relevant predictors for both suicide attempts and suicide death. These same associations were previously reported in several meta-analyses [12, 13, 56]. The different follow-up periods of the cohort studies included in this review imply that a history of suicide attempts may be a strong predictor of future suicidal behaviour at both the short and long term (6 weeks to 10 years). Conversely, history of suicide attempts alone does not appear to be a relevant predictor for suicidal ideation, which is consistent with previous results found in adults with a history of suicide attempt [57].

For the first time, suicidal ideation and severity of suicidal ideation have been meta-analysed as predictors of suicidal behaviours in patients with depressive disorders. Our findings suggest that suicidal ideation is a relevant predictor of subsequent suicide behaviours, which is in line with previous studies conducted in patients with depression [58–61]. We hypothesize that the high heterogeneity found in the severity of suicidal ideation predictor could be due to the different setting in which the patients were recruited by the pooled studies. A closer analysis revealed that the study conducted in primary care patients [22] presented lower levels of suicidal ideation than those performed in hospital settings [18, 21]. When the study conducted in primary care was excluded, subsequent analysis indicated that the severity of suicidal ideation was a significant predictor of suicide attempts (OR_w_ = 5.80; 95% CI 3.26–10.30). Therefore, to estimate the predictive ability of the severity of suicidal ideation, the clinical setting of the patients may be considered.

Depression severity emerged in this review as a statistically relevant predictor for future suicide attempts and suicide death, but not for suicidal ideation. Severity of depression has been previously found to be a mediator of suicidal ideation [62]. In addition, it has been detected that decreased levels of suicidal ideation are preceded by a decrease in the severity of depression [17]. These findings could not be corroborated in this review due to the limited number of studies. Further elucidation of the dynamics between suicidal ideation and depression severity is required. However, our results regarding suicidal behaviours outcomes are in line with those found in previous meta-analyses [12, 13]. Recently, it has been observed that both severity and variability of depression consistently predicted future suicide attempts [63]. Therefore, understanding which specific parameters of suicidal ideation and depression (e.g., intensity, severity, variability, duration, frequency) best predict suicide risk arise as an important goal of future research.

Consistent with recent studies [64–66] and meta-analysis [67], sleep disturbances were identified as predictors of suicide death. Although studies pooled in the meta-analysis performed by Hawton et al. [12] and this review are almost the same [29, 44] for this predictor, results obtained are not consistent. Differences in data extraction (e.g., raw data vs computed OR) could explain this discrepancy. Other clinical predictors such as psychotic features have been associated in this review with a higher risk of suicide attempts and suicide death. This relationship was stated by previous longitudinal studies [68] and meta-analysis [69], with reported ORs similar to those found in this review (OR_w_ = 1.57).

Older age of diagnosis was associated in this review with a greater risk of suicide death. This may be, however, a spurious relationship given that pooled results were not controlled for the age variable and it is known that the risk of suicide increases with age [70]. Interestingly, and despite few evidence (*k* = 2), more time to full remission of MDE was identified as a predictor for future suicide attempts. To determine when exactly patients with depression are at highest risk of suicide is highly relevant to prevent suicide [31, 71, 72]. One recent study showed that suicidal ideation persist during remission of MDD [73], suggesting that the risk of suicide may not end with the decline of depressive symptoms. If these results were corroborated by other studies, it would indicate that the assessment of suicide risk in people with depression should be extended beyond the remission of the depressive episode.

Among those predictors that could not be meta-analysed because there was only a single prediction case supporting them (*k* = 1), those that significantly predicted a greater risk of future suicide attempts were the number of previous suicides attempts and having a diagnosis of severe depression. Similarly, those that significantly predicted an increased risk of suicide death were suicidal planning, having a decreased appetite, pessimism, or having depressive symptoms during follow-up. There were no relevant predictors of suicidal ideation other than those already reported in the meta-analysis section. Contrasting the robustness of these clinical predictors across other longitudinal studies would be of great interest for exploring potentially relevant risk factors associated with suicidality within depression.

### Study limitations

This review is not without limitations. First, seeking for greater conceptual and methodological consistency among potentially included studies, the search strategy was limited to literature in English published from 2001 to 2022. This approach implied, however, a selection bias [74], as not all studies conducted to date were reviewed. Second, the included studies were restricted to adult patients, so results cannot be extended to children or youths with depressive disorders. A recent systematic review conducted by Moller et al. [75] reported depression predictors, psychiatric comorbidities and neurological predictors of suicidality in youth patients with depressive disorders. Third, we found that differences in sample source, depressive diagnoses, cohort follow-up, and suicidality outcome inventories integrated in this review are highly heterogeneous across the studies. Therefore, the lack of subgroups analyses is deemed an important limitation of this review. Subgroup analyses were not performed considering sex differences, so it cannot be concluded that the reported predictors are equally robust for both sexes. The exclusion of studies with a very small sample size (*N* ≤ 20) was intended to include only those studies with minimally precise estimates. This decision precludes, however, comparison between our results and those of studies with very small samples. The few quantitative data obtained from the studies hindered us from meta-analyse both adjusted and unadjusted results. The number of prediction cases for each predictor was also insufficient to reliably detect publication bias, a significant requirement for contrasting the validity of results in meta-analyses. Additionally, the NOS S4 criterion inadequately screens outcomes of an episodic nature, such as suicidal behaviour. As a result, an adjustment of this criterion was required to suit this specific circumstance. Furthermore, the fluctuating nature of suicidality has prompted the use of advanced statistical analysis techniques (e.g., multilevel mixed models), which are better adapted to analyse multiple repeated measures. This is especially noticeable for the suicidal ideation outcome. Future meta-analyses in this field should consider these new results and the variability derived from these different methods of analysis.

## Conclusions

After 10 years of the first systematic review on suicide predictors in patients with depressive disorders [12], one of the most striking finding remains the paucity of studies reporting depression-related predictors for suicidality. The maximum number of studies supporting a predictor is inadequate (*k* = 5). Moreover, only one study was conducted exclusively in primary care centres, which is particularly alarming if we consider that it is in primary care centres where patients seek help most frequently in the months prior to suicide [76]. Nevertheless, this review enables to pinpoint which characteristics of depression a clinician should focus on when assessing the risk of suicide in a patient with depression.

Previous suicide attempts and suicidal ideation, more severe depression and hopelessness, presence of psychotic symptoms, sleep disturbances, and longer time to full remission emerged as the main clinical predictors for suicidality in adults with depression. Although not enough studies have yet been conducted to comprehensively analyse the relationship between the depression core symptoms (e.g., anhedonia, sadness, loss of concentration) and clinical course features (e.g., recurrence, duration) and subsequent suicidality, our results showed that within the clinical picture of depression there is not one predictor category (i.e., diagnostic subtype, clinical symptoms, clinical course, clinical assessment scales) more striking than others in predicting suicidality, but all should be considered for adequate suicide prevention. Further studies are needed to corroborate these results, to clarify contradictory findings, to examine specific interactions and temporal dynamics between predictors and suicidal outcomes, and to explore more robust clinical predictors of suicidality.

## Supplementary Information

Below is the link to the electronic supplementary material.

Supplementary file1 (DOCX 1279 KB)

## Data Availability

Data supporting the results of this study can be requested by e-mail from the corresponding author.
