# Supplementary material for: Clinical predictors of suicidal ideation, suicide attempts and suicide death in depressive disorder: a systematic review and meta-analysis
Source: Eur Arch Psychiatry Clin Neurosci. 2023 Nov 28;274(7):1543–63. doi: 10.1007/s00406-023-01716-5 (PMC11422269; doi:10.1007/s00406-023-01716-5)
Supplement: Supplementary file 1 — Supplementary file1 (DOCX 1279 KB) [file 406_2023_1716_MOESM1_ESM.docx]

**Supplementary material**

**Title:** Clinical predictors of suicidal ideation, suicide attempts and suicide death in depressive disorder: a systematic review and meta-analysis

**Authors:** Pau Riera-Serra, Guillem Navarra-Ventura, Adoración Castro, Margalida Gili, Angie Salazar-Cedillo, Ignacio Ricci-Cabello, Lorenzo Roldán-Espínola, Victoria Coronado-Simsic, Mauro García-Toro, Rocío Gómez-Juanes, Miquel Roca

**Corresponding author:** Adoración Castro PhD; Research Institute of Health Sciences (IUNICS), University of the Balearic Islands (UIB); Carretera de Valldemossa, km 7.5, 07122 Palma de Mallorca, Balearic Islands, Spain

E-mail: [a.castro@uib.es](mailto:a.castro@uib.es)

Phone: +34 971 25 98 88 (ext: 9888)

**Journal:** *European Archives of Psychiatry and Clinical Neuroscience*

| **Table S1.** Search strategy: domains and keywords | |
| --- | --- |
| **Domains** | **Keywords** |
| Depression | Depression  Depressive disorder  Major depression  Dysthymia |
| Suicide | Suicide  Suicidality  Suicidal behaviour  Suicide attempt  Suicide ideation  Suicide thoughts  Suicide death  Suicide plan  Suicide threat  Self-injury  Self-harm  Self-mutilation  Self-cutting  Self-poisoning  Self-destructive |
| Study design | Case control study  Cohort study  Cohort analysis  Follow up study  Retrospective  Longitudinal  Longitudinally  Prospective  Prospectively |
| Filters | Humans ≥ 18 years old (adults) English From 1^st^ January 2001 |

| **Table S2.** Pubmed (Medline) search strategy: queries and results (searched on October 3, 2022) | | |
| --- | --- | --- |
| **N₀** | **Queries** | **Results** |
| #1 | "depression"[MeSH Major Topic] | 66,767 |
| #2 | "depressive disorder"[MeSH Major Topic] | 94,333 |
| #3 | "depress*"[Title/Abstract] | 539,169 |
| #4 | "major depressive disorder"[Title/Abstract] | 29,445 |
| #5 | "MDD"[Title/Abstract] | 17,117 |
| #6 | "major depression"[Title/Abstract] | 25,963 |
| #7 | "dysthymi*"[Title] | 627 |
| #8 | "dysthymia"[Title/Abstract] | 2,286 |
| #9 | "suicide"[MeSH Major Topic] | 52,697 |
| #10 | "self mutilation"[MeSH Major Topic] | 2,270 |
| #11 | "suicid*"[Title/Abstract] | 91,689 |
| #12 | "self injur*"[Title/Abstract] | 6,042 |
| #13 | "self harm*"[Title/Abstract] | 8,078 |
| #14 | "self mutilat*"[Title/Abstract] | 1,889 |
| #15 | "self cut*"[Title/Abstract] | 233 |
| #16 | "self destruct*"[Title/Abstract] | 2,124 |
| #17 | "case control studies"[MeSH Major Topic] | 1,856 |
| #18 | "cohort studies"[MeSH Major Topic] | 4,604 |
| #19 | "retrospective*"[Title/Abstract] | 952,805 |
| #20 | "longitudinal*"[Title/Abstract] | 325,980 |
| #21 | "follow up stud*"[Title/Abstract] | 56,709 |
| #22 | "prospective*"[Title/Abstract] | 827,433 |
| #23 | "cohort stud*"[Title/Abstract] | 292,112 |
| #24 | "case-control*"[Title/Abstract] | 152,204 |
| #25 | #1 OR #2 OR #3 OR #4 OR #5 OR #6 OR #7 OR #8 | 227,858 |
| #26 | #9 OR #10 OR #11 OR #12 OR #13 OR #14 OR #15 OR #16 | 108,404 |
| #27 | #17 OR #18 OR #19 OR #20 OR #21 OR #22 OR #23 OR #24 | 2,208,913 |
| #28 | #25 AND #26 AND #27 | 2,109 |
| #29 | #28 AND ((humans[Filter]) AND (2001/1/1:2022/10/03[pdat]) AND (english[Filter]) AND (alladult[Filter])) | 1,162 |

| **Table S3.** EMBASE search strategy: queries and results (searched on October 3, 2022) | | |
| --- | --- | --- |
| **N₀** | **Queries** | **Results** |
| #1 | 'major depression'/exp | 71329 |
| #2 | 'mdd':ab,ti | 22282 |
| #3 | 'depress*':ab,ti | 357921 |
| #4 | depress*:ti | 208736 |
| #5 | 'major depression':ab,ti | 32439 |
| #6 | 'major depressive disorder':ab,ti | 35125 |
| #7 | dysthymi*:ti | 793 |
| #8 | dysthymia:ab,ti | 2914 |
| #9 | 'suicide'/exp | 63022 |
| #10 | suicid*:ab,ti | 108359 |
| #11 | 'self injur*':ab,ti | 6526 |
| #12 | 'self harm*':ab,ti | 8701 |
| #13 | 'self mutilat*':ab,ti | 2131 |
| #14 | 'self cut*':ab,ti | 291 |
| #15 | 'self destruct*':ab,ti | 2506 |
| #16 | 'automutilation'/exp | 21102 |
| #17 | 'case control study'/exp | 194577 |
| #18 | 'cohort analysis'/exp | 748338 |
| #19 | retrospective*:ab,ti | 1407553 |
| #20 | longitudinal*:ab,ti | 395769 |
| #21 | 'follow-up stud*':ab,ti | 69444 |
| #22 | prospective*:ab,ti | 1180851 |
| #23 | 'cohort stud*':ab,ti | 357168 |
| #24 | 'case-control*':ab,ti | 175709 |
| #25 | 'cohort analy*':ab,ti | 15062 |
| #27 | #1 OR #2 OR #3 OR #4 OR #5 OR #6 OR #7 OR #8 | 412236 |
| #28 | #9 OR #10 OR #11 OR #12 OR #13 OR #14 OR #15 OR #16 | 142282 |
| #29 | #17 OR #18 OR #19 OR #20 OR #21 OR #22 OR #23 OR #24 OR #25 | 3405532 |
| #30 | #26 AND #27 AND #28 AND ([adult]/lim OR [aged]/lim) AND [humans]/lim AND [english]/lim AND [embase]/lim AND [2001-2022]/py | 3411 |

| **Table S4.** PsychINFO search strategy: queries and results (searched on October 3, 2022) | | |
| --- | --- | --- |
| **N₀** | **Queries** | **Results** |
| #1 | DE major depression | 142408 |
| #2 | DE depression | 51159 |
| #3 | MA depressive disorder | 60319 |
| #4 | MA depression | 55734 |
| #5 | TI depress* | 127296 |
| #6 | TI "major depressive disorder" OR AB "major depressive disorder" | 23413 |
| #7 | TI "MDD" OR AB "MDD" | 12872 |
| #8 | TI "major depression" OR AB "major depression" | 23643 |
| #9 | TI dysthymi* | 647 |
| #10 | TI dysthymia AND AB dysthymia | 315 |
| #11 | MA suicide | 22490 |
| #12 | DE suicide | 35428 |
| #13 | MA self-injurious behavior | 4120 |
| #14 | DE self-injurious behavior | 7920 |
| #15 | TI suicid* OR AB suicid* | 72523 |
| #16 | TI self-injur* OR AB self-injur* | 3618 |
| #17 | TI self-harm* OR AB self-harm* | 6817 |
| #18 | TI self-mutilat* AND AB self-mutilat* | 458 |
| #19 | TI self-cut* AND AB self-cut* | 73 |
| #20 | TI self-destruct* AND AB self-destruct* | 311 |
| #21 | DE cohort analysis | 1651 |
| #22 | TI "cohort stud*" OR AB "cohort stud*" | 26522 |
| #23 | TI "case-control*" OR AB "case-control*" | 12679 |
| #24 | TI "cohort analy*" OR AB "cohort analy*" | 901 |
| #25 | TI retrospective* OR AB retrospective* | 50214 |
| #26 | TI longitudinal* OR AB longitudinal* | 136781 |
| #27 | TI "follow-up stud*" OR AB "follow-up stud*" | 13518 |
| #28 | TI prospective* OR AB prospective* | 77389 |
| #29 | S1 OR S2 OR S3 OR S4 OR S5 OR S6 OR S7 OR S8 OR S9 ORS 10 | 220919 |
| #30 | S11 OR S12 OR S13 OR S14 OR S15 OR S16 OR S17 OR S18 OR S19 OR S20 | 81913 |
| #31 | S21 OR S22 OR S23 OR S24 OR S25 OR S26 OR S27 OR S28 | 273958 |
| #32 | S29 AND S30 AND S31 | 2349 |
| #33 | S29 AND S30 AND S31  ( Limiters - Publication date: 2001-01-01-2022-10-31; Language: English; Age group: Adulthood (18 yrs & older); Population group: Human). | 1463 |

| **Table S5.** Cochrane search strategy: queries and results (searched on October 3, 2022) | | |
| --- | --- | --- |
| **N₀** | **Queries** | **Results** |
| #1 | MeSH descriptor: [Depression] 1 tree(s) exploded | 14235 |
| #2 | MeSH descriptor: [Depressive Disorder] explode all trees | 13449 |
| #3 | (depress*):ti | 33986 |
| #4 | (“major depressive disorder”):ti OR (“major depressive disorder”):ab | 7866 |
| #5 | (“MDD”):ti OR (“MDD”):ab | 4225 |
| #6 | ("major depression"):ti OR ("major depression"):ab | 5971 |
| #7 | (dysthymi*):ti | 198 |
| #8 | (dysthymia):ti OR (dysthymia):ab | 479 |
| #9 | MeSH descriptor: [Suicide] explode all trees | 1509 |
| #10 | MeSH descriptor: [Self Mutilation] explode all trees | 37 |
| #11 | (suicid*):ti OR (suicid*):ab | 6477 |
| #12 | (self-injur*):ti OR (self-injur*):ab | 517 |
| #13 | (self-harm*):ti OR (self-harm*):ab | 774 |
| #14 | (self-mutilat*):ti OR (self-mutilat*):ab | 34 |
| #15 | (self-cut*):ti OR (self-cut*):ab | 15 |
| #16 | (self-destruct*):ti OR (self-destruct*):ab | 51 |
| #17 | MeSH descriptor: [Case-Control Studies] explode all trees | 15135 |
| #18 | MeSH descriptor: [Cohort Studies] explode all trees | 161158 |
| #19 | ("Case-control*"):ti OR ("Case-control*"):ab | 5117 |
| #20 | ("Cohort stud*"):ti OR ("Cohort stud*"):ab | 0 |
| #21 | ("Cohort analy*"):ti OR ("Cohort analy*"):ab | 0 |
| #22 | (Retrospective*):ti AND (Retrospective*):ab | 1903 |
| #23 | (Longitudinal*):ti OR (Longitudinal*):ab | 20305 |
| #24 | ("Follow-up stud*"):ti OR ("Follow-up stud*"):ab | 0 |
| #25 | (Prospective*):ti OR (Prospective*):ab | 211230 |
| #26 | #1 OR #2 OR #3 OR #4 OR #5 OR #6 OR #7 OR #8 | 45117 |
| #27 | #9 OR #10 OR #11 OR #12 OR #13 OR #14 OR #15 OR #16 | 7177 |
| #28 | #17 OR #18 OR #19 OR #20 OR #21 OR #22 OR #23 OR #24 OR #25 | 318073 |
| #29 | #26 AND #27 AND #28 with Cochrane Library publication date Between Jan 2001 and Oct 2022 | 307 |

| **Table S6.** OATD search strategy: queries and results (searched on October 3, 2022) | | |
| --- | --- | --- |
| **N₀** | **Queries** | **Results** |
| #1 | title: depress* | 14183 |
| #2 | title:("major depressive disorder") OR abstract:("major depressive disorder") | 1528 |
| #3 | title:( “major depression” ) OR abstract:( “major depression” ) | 331925 |
| #4 | title:( dysthym* ) OR abstract: ( dysthym* ) | 149 |
| #5 | title:( Suicid* ) OR abstract:( Suicid* ) | 9435 |
| #6 | title:( self-injur* ) OR abstract:( self-injur* ) | 0 |
| #7 | title:( "self-harm*" ) OR abstract:( "self-harm*" ) | 977 |
| #8 | title:( self-mutilat*) OR abstract:( self-mutilat* ) | 0 |
| #9 | title:( self-cut* ) OR abstract:( self-cut* ) | 0 |
| #10 | title:( "self-destruct*" ) OR abstract:( "self-destruct*" ) | 2 |
| #11 | title:( "case-control*" ) OR abstract:( "case-control*" ) | 5367 |
| #12 | title:( "cohort stud*" ) OR abstract:( "cohort stud*" ) | 0 |
| #13 | title:( "cohort analy*" ) OR abstract:( "cohort analy*" ) | 0 |
| #14 | title:( retrospective* ) OR abstract:( retrospective* ) | 23819 |
| #15 | title:( longitudinal* ) OR abstract:( longitudinal* ) | 53903 |
| #16 | title:( "follow-up stud*" ) OR abstract:( "follow-up stud*") | 0 |
| #17 | title:( prospective* ) OR abstract:( prospective* ) | 29311 |
| #18 | title:("MDD") OR abstract:("MDD") | 1268 |
| #19 | title:("MDD") OR abstract:("MDD") OR (title:( “major depression” ) OR abstract:( “major depression” )) OR (title: depress*) OR (title:( “major depression” ) OR abstract:( “major depression” )) OR (title:( dysthym* ) OR(abstract: ( dysthym* ) ) | 334987 |
| #20 | (title:( Suicid* ) OR abstract:( Suicid* )) OR (title:( "self-injur*" ) OR abstract:( "self-injur*" )) OR (title:( "self-harm*" ) OR abstract:( "self-harm*" )) OR (title:( self-mutilat*) OR abstract:( self-mutilat* )) | 9952 |
| #21 | title:( "Case-control*" ) OR abstract:( "Case-control*" ) OR title:( "Cohort stud*" ) OR abstract:( "Cohort stud*" ) OR title:( "Cohort analy*" ) OR abstract:( "Cohort analy*" ) OR (title:( Retrospective* ) OR abstract:( Retrospective* )) OR (title:( Longitudinal* ) OR abstract:( Longitudinal* )) OR title:( "Follow-up stud*" ) OR abstract:( "Follow-up stud*") OR title:( Prospective* ) OR abstract:( Prospective* ) | 104667 |
| #22 | ((title:("MDD") OR abstract:("MDD")) OR (title:( “major depression” ) OR abstract:( “major depression” )) OR (title: depress*) OR (title:( “major depression” ) OR abstract:( “major depression” )) OR (title:( dysthym*) OR (abstract: ( dysthym* ))) AND ((title:( Suicid* ) OR abstract:( Suicid* )) OR (title:( "self-injur*" ) OR abstract:( "self-injur*" )) OR (title:( "self-harm*" ) OR abstract:( "self-harm*" )) OR (title:( self-mutilat*) OR abstract:( self-mutilat* ))) AND (title:( "Case-control*" ) OR abstract:( "Case-control*" ) OR title:( "Cohort stud*" ) OR abstract:( "Cohort stud*" ) OR title:( "Cohort analy*" ) OR abstract:( "Cohort analy*" ) OR (title:( Retrospective* ) OR abstract:( Retrospective* )) OR (title:( Longitudinal* ) OR abstract:( Longitudinal* )) OR title:( "Follow-up stud*" ) OR abstract:( "Follow-up stud*") OR title:( Prospective* ) OR abstract:( Prospective* )) | 278 |
| #23 | ((title:("MDD") OR abstract:("MDD")) OR (title:( “major depression” ) OR abstract:( “major depression” )) OR (title: depress*) OR (title:( “major depression” ) OR abstract:( “major depression” )) OR (title:( dysthym*) OR(abstract: ( dysthym* ))) AND ((title:( Suicid* ) OR abstract:( Suicid* )) OR (title:( "self-injur*" ) OR abstract:( "self-injur*" )) OR (title:( "self-harm*" ) OR abstract:( "self-harm*" )) OR (title:( self-mutilat*) OR abstract:( self-mutilat* ))) AND (title:( "Case-control*" ) OR abstract:( "Case-control*" ) OR title:( "Cohort stud*" ) OR abstract:( "Cohort stud*" ) OR title:( "Cohort analy*" ) OR abstract:( "Cohort analy*" ) OR (title:( Retrospective* ) OR abstract:( Retrospective* )) OR (title:( Longitudinal* ) OR abstract:( Longitudinal* )) OR title:( "Follow-up stud*" ) OR abstract:( "Follow-up stud*") OR title:( Prospective* ) OR abstract:( Prospective* )) + filter (English) + filter (date: 2001-2022) | 155 |

| **Table S7**. WoS Science Citation Index search strategy: queries and results (searched on October 3, 2022) | | |
| --- | --- | --- |
| **N₀** | **Queries** | **Results** |
| #1 | (TI= “depress*”) AND LA=(English) | 25767 |
| #2 | (TS= “MDD”) AND LA=English | 1670 |
| #3 | (TS= “major depressive disorder”) AND LA= English | 2766 |
| #4 | (TS= “major depression”) AND LA= English | 3653 |
| #5 | (TI= “ dysthymi*”) AND LA= English | 75 |
| #6 | (TS= “ dysthymia”) AND LA= English | 156 |
| #7 | (TS= “suicid*”) AND LA= English | 6165 |
| #8 | (TS= “self-injur*”) AND LA= English | 291 |
| #9 | (TS= “self-harm*”) AND LA= English | 235 |
| #10 | (TS= “ self-mutilat*”) AND LA= English | 69 |
| #11 | (TS= " self-cut*") AND LA= English | 11 |
| #12 | (TS= " self-destruct*") AND LA= English | 270 |
| #13 | (TS= "Case-control*") AND LA= English | 8248 |
| #14 | (TS= "Cohort stud*") AND LA= English | 16426 |
| #15 | (TS= "Cohort analy*") AND LA= English | 977 |
| #16 | (TS= “ Retrospective* “) AND LA= English | 57914 |
| #17 | (TS= “ Longitudinal* “) AND LA= English | 61675 |
| #18 | (TS= “ Prospective* “) AND LA= English | 72337 |
| #19 | (TS= "Follow-up stud*") AND LA= English | 5172 |
| #20 | #6 OR #5 OR #4 OR #3 OR #2 OR #1 | 28278 |
| #21 | #12 OR #11 OR #10 OR #9 OR #8 OR #7 | 6763 |
| #22 | #19 OR #18 OR #17 OR #16 OR #15 OR #14 OR #13 | 204884 |
| #23 | #20 AND #21 AND #22 | 53 |

| **Table S8**. Opengrey search strategy: queries and results (searched on October 3, 2022) | | |
| --- | --- | --- |
| **N₀** | **Queries** | **Results** |
| #1 | depress* | 3176 |
| #2 | suicid* | 999 |
| #3 | depress* AND suicid | 151 |
| #4 | depress* AND suicid* lang:"en" | 26 |
| #5 | (>=2001) | 16 |

| **Table S9.** Classification of the predictors of suicidality within depression found among the included studies^a^ | | | |
| --- | --- | --- | --- |
| **Diagnostic subtype** (n = 5; *k* = 19)^b^ | **Clinical symptoms** (n = 23; *k* = 31)^b^ | **Clinical course**  (n = 10; *k* = 35)^b^ | **Clinical assessment scales**  (n = 7; *k* = 28)^b^ |
| Atypical features (1) | Agitation (1) | Age at diagnosis of MDD (5) | Beck Depression Inventory (BDI) (8) |
| Dysthymia (1) | Anhedonia (1) | Family history of suicide or suicide attempts (1) | Beck Hopelessness Scale (BHS) (8) |
| Melancholic subtype (4) | Appetite gain (1) | Number of major depressive episodes (5) | Beck Scale for Suicide Ideation (BSSI) (5) |
| Psychotic subtype (7) | Decreased appetite (1) | Number of suicide attempts (1) | Hamilton Depression Rating Scale (HDRS) (3) |
| Severe depression (6) | Depressed mood (1) | Personal history of depression (1) | Hospital Anxiety and Depression Scale, depression subscale (HADS-D) (1) |
| - | Depression Symptomatology (1) | Personal history of suicide attempt (11) | Inventory of Depressive Symptomatology (IDS) (1) |
| - | Fatigue (1) | Personal history of suicidal ideation (3) | Montgomery-Åsberg Depression Rating Scale (MADRS) (2) |
| - | Guilt (2) | Time to full remission (3) | - |
| - | Hopelessness (2) | Total time in depression (4) | - |
| - | Hypersomnia (1) | Time since depression diagnosis (1) | - |
| - | Insomnia (2) | - | - |
| - | Loss of concentration (1) | - | - |
| - | Persistent sleep disturbance (1) | - | - |
| - | Pessimism (1) | - | - |
| - | Psychomotor disturbance (1) | - | - |
| - | Psychotic symptoms (2) | - | - |
| - | Reduced sleep (1) | - | - |
| - | Retardation (1) | - | - |
| - | Sexual interest (1) | - | - |
| - | Sleep complaints (2) | - | - |
| - | Suicidal ideation (4) | - | - |
| - | Suicide plans (1) | - | - |
| - | Weight loss (1) | - | - |
| ^a^ The number of prediction cases (*k)* for each predictor are reported in parentheses. Predictors for which we found more than one prediction case (*k > 1*) were eventually meta-analysed.  ^b^ Number of predictors (n) and prediction cases (*k*) for each predictor category.  - Indicates that no more predictors were found for the predictor category. | | | |

| **Table S10.** Pre-operationalization of several predictors prior to the meta-analysis | | |
| --- | --- | --- |
| **Generated predictors** | **Analogous predictors** | **Relabelled predictors** |
| Severity of depression (15) | Beck Depression Inventory (BDI) (8)  Hamilton Depression Rating Scale (HDRS) (3) Hospital Anxiety and Depression Scale, depression subscale (HADS-D) (1) Montgomery-Åsberg Depression Rating Scale (MADRS) (2) Inventory of Depressive Symptomatology (IDS) (1) | - |
|  |  |  |
| Suicidal ideation (7) | Personal history of suicidal ideation (3)  Suicidal ideation (4) | - |
|  |  |  |
| Severity of suicidal ideation (5) | - | Beck Scale for Suicide Ideation (BSSI) (5) |
|  |  |  |
| Severity of hopelessness (8) | - | Beck Hopelessness Scale (BHS) (8) |
|  |  |  |
| Psychotic features (9) | Psychotic subtype (7) Psychotic symptoms (2) | - |
|  |  |  |
| Sleep disturbances (7) | Hypersomnia (1)  Insomnia (2)  Persistent sleep disturbance (1)  Reduced sleep (1)  Sleep complaints (2) | - |
| ^a^ Number of prediction cases (*k)* found for each predictor are reported in parentheses. | | |

| **Table S11.** Newcastle-Ottawa quality assessment of the included studies in the systematic review | | | | | | | | | | | | |
| --- | --- | --- | --- | --- | --- | --- | --- | --- | --- | --- | --- | --- |
| N₀ | Study | Design | Suicide outcome | Selection | | | | Comparability | Outcome | | | NOS score |
|  |  |  |  | S1 | S2^a^ | S3 | S4^b^ | C1 | E/O1 | E/O2^c^ | E/O3^d^ |  |
| 1 | Aaltonen *et al.* (2019) | cohorts | death | ★ | ★ | ★ | ★ | ★★ | ★ | ★ | - | 8 |
| 2 | Baryshnikov *et al.* (2020) | cohorts | ideation | ★ | ★ | ★ | - | ★★ | - | ★ | ★ | 7 |
| 3 | Chan *et al.* (2014) | cohorts | attempt | - | ★ | ★ | - | ★★ | - | ★ | ★ | 6 |
| 4.1 | Courtet *et al.* (2014) | cohorts | ideation | ★ | ★ | ★ | - | ★★ | - | ★ | ★ | 7 |
| 4.2 | Courtet *et al.* (2014) | cohorts | attempt | ★ | ★ | ★ | - | ★★ | - | ★ | - | 6 |
| 5 | Galfalvy *et al.* (2008); Grunebaum *et al.* (2004) | cohort | attempt | ★ | ★ | ★ | - | ★★ | - | ★ | ★ | 7 |
| 6 | Gladstone *et al.* (2001) | cohort | death | ★ | ★ | - | - | ★ | ★ | ★ | - | 5 |
| 7 | Gronemann *et al.* (2021); Kessing (2004) | cohort | death | ★ | ★ | ★ | ★ | ★★ | ★ | ★ | ★ | 9 |
| 8.1 | Sokero *et al.* (2006) | cohort | ideation | ★ | ★ | ★ | - | ★★ | - | ★ | ★ | 7 |
| 8.2 | Holma *et al.* (2010); Sokero *et al.* (2005) | cohort | attempt | ★ | ★ | ★ | - | ★★ | - | ★ | ★ | 7 |
| 9 | Kim *et al.* (2012) | case-control | death | - | ★ | ★ | ★ | ★★ | ★ | ★ | ★ | 8 |
| 10 | Leadholm *et al.* (2014) | cohort | death | ★ | ★ | ★ | ★ | ★★ | ★ | - | - | 7 |
| 11 | Manning *et al.* (2021) | cohort | ideation | ★ | ★ | ★ | - | ★★ | ★ | ★ | ★ | 8 |
| 12 | McGirr *et al.* (2007); McGirr *et al.* (2008) | case-control | death | ★ | ★ | - | - | ★ | ★ | ★ | ★ | 6 |
| 13 | Nikel *et al.* (2006) | cohort | attempt | ★ | ★ | - | - | ★ | - | ★ | ★ | 5 |
| 14 | Nobile *et al.* (2022) | cohort | attempt | ★ | ★ | - | - | ★★ | ★ | ★ | ★ | 7 |
| 15.1 | Qiu *et al.* (2017) | cohort | ideation | ★ | ★ | ★ | - | - | - | ★ | ★ | 5 |
| 15.2 | Qiu *et al.* (2017) | cohort | attempt | ★ | ★ | ★ | - | - | - | ★ | ★ | 5 |
| 16 | Reutfors *et al.* (2021) | case-control | death | ★ | ★ | ★ | - | ★★ | ★ | ★ | ★ | 8 |
| 17 | Riihimäki *et al.* (2014) | cohort | attempt | ★ | ★ | ★ | - | ★★ | - | ★ | ★ | 7 |
| 18 | Rönnqvist *et al.* (2021) | cohort | death | ★ | ★ | ★ | - | ★★ | ★ | ★ | - | 7 |
| 19 | Schneider *et al.* (2001) | cohort | death | ★ | ★ | ★ | - | - | - | ★ | ★ | 5 |
| NOS: Newcastle-Otawa Scale  S1: In cohort studies: “*Representativeness of the exposed cohort*”; In case-control studies: “*Is the case definition adequate?*”  S2: In cohort studies: “*Selection of the non-exposed cohort*”; In case-control studies: “*Representativeness of the cases”.* S3: In cohort studies: “*Ascertainment of exposure*”; In case-control studies: “*Selection of controls”.* S4: In cohort studies: “*Demonstration that outcome of interest was not present at start of study*”; In case-control studies: “*Definition of controls”.*  C1: In cohort studies: “*Comparability of cohorts on the basis of the design or analysis*”; In case-control studies: “*Comparability of cases and controls on the basis of the design or analysis”.*  E/O1: In cohort studies: “*Assessment of outcome*”; In case-control studies: “*Ascertainment of exposure”.*  E/O2: In cohort studies: “*Was follow-up long enough for outcomes to occur*”; In case-control studies: *“Same method of ascertainment for cases and controls”.*  E/O3: In cohort studies: “*Adequacy of follow up cohorts*”; In case-control studies: “*Non-response rate”.*  ^a^ S2 criterion was always evaluated positively in cohort studies since the exposure factors under study (i.e., clinical predictors of suicidality) were always assessed at baseline from a single clinical population.  ^b^ S4 criterion was scored positively when no more than 10% of study participants had a history of the suicidal outcomes at baseline.  ^c^ In cohorts studies the item of the NOS “Outcome” dimension (“Was the follow-up long enough for outcomes to occur”) was considered adequate when follow-up for suicidal ideation was at least 1 month, for suicide attempts was at least 6 months and for suicidal completers was at least 12 months. ^d^ In cohorts studies the item of the NOS “Outcome” dimension ( “Adequacy of the follow-up of cohorts”) was deemed adequate when subjects lost to follow-up were ≤ 30%. | | | | | | | | | | | | |

| **Table S12.**  Meta-analysis of hazard ratios for depression-related predictors of suicide attempts and suicide death | | | | | | |
| --- | --- | --- | --- | --- | --- | --- |
| Outcome | Clinical Predictors | Study (year) | HR (95 % CI) | Weight | wHR (95 % CI) | Heterogeneity |
| Suicide  attempts | History of suicide attempt | Galfalvy et al. (2008) | 5.14 (2.27 – 11.65) | 54.83 % | 4.79 (2.61 – 8.77) *P* < 0.001 | I² = 0 % *χ² =* 0.06; *P* = 0.800 |
|  |  | Holma et al. (2010) | 4.39 (1.78 – 10.80) | 45.17 % |  |  |
|  |  |  |  |  |  |  |
|  | Severity of depression | Galfalvy et al. (2008) | 1.01 (0.97 – 1.05) | 99.06 % | 1.03 (0.98 – 1.08)  *P* = 0.219 | I² = 0 % *χ² =* 0.04; *P* = 0.851 |
|  |  | Nobile et al. (2022) | 1.08 (0.66 – 1.77) | 0.94 % |  |  |
|  |  |  |  |  |  |  |
|  | Severity of suicidal ideation | Galfalvy et al. (2008) | 1.06 (1.03 – 1.09) | 55.33 % | 1.59 (0.66 – 3.86)  *P* = 0.304 | I² = 89.30 % *χ² =* 9.33; *P* = 0.002 |
|  |  | Nobile et al. (2022) | 2.63 (1.36 – 4.36) | 44.67 % |  |  |
| Suicide death | Severity of depression | Aaltonen et al. (2019) | 1.19 (1.08 – 1.30) | 85.91 % | 1.21 (1.11 – 1.32)  *P* < 0.001 | I² = 0 % *χ² =* 1.94; *P* = 0.379 |
|  |  | Gronemann *et al.* (2021) | 1.52 (1.07 – 2.15) | 5.98 % |  |  |
|  |  | Ronnqvist et al. (2021) | 1.28 (0.95 – 1.73) | 8.11 % |  |  |
|  |  |  |  |  |  |  |
|  | Psychotic  features | Aaltonen et al. (2019) | 1.38 (1.24 – 1.53) | 93.09 % | 1.36 (1.22 – 1.51)  *P* < 0.001 | I² = 0 % *χ² =* 0.66; *P* = 0.416 |
|  |  | Ronnqvist et al. (2021) | 1.16 (0.78 – 1.72) | 6.91 % |  |  |
| HR: Hazard Ratio; wHR: weighted Hazard Ratio. | | | | | | |


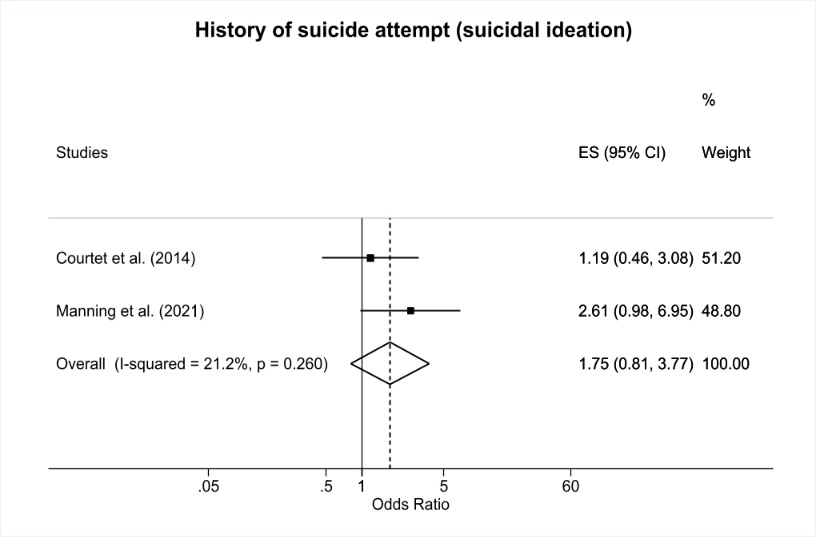

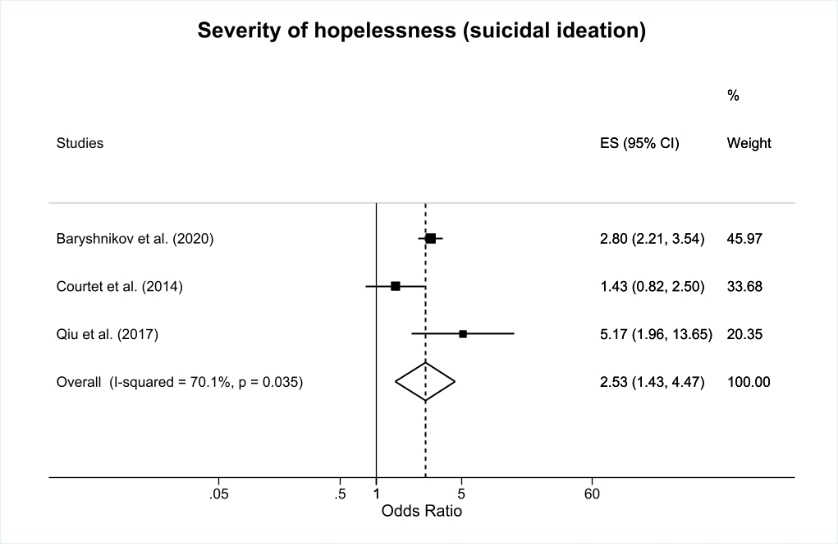


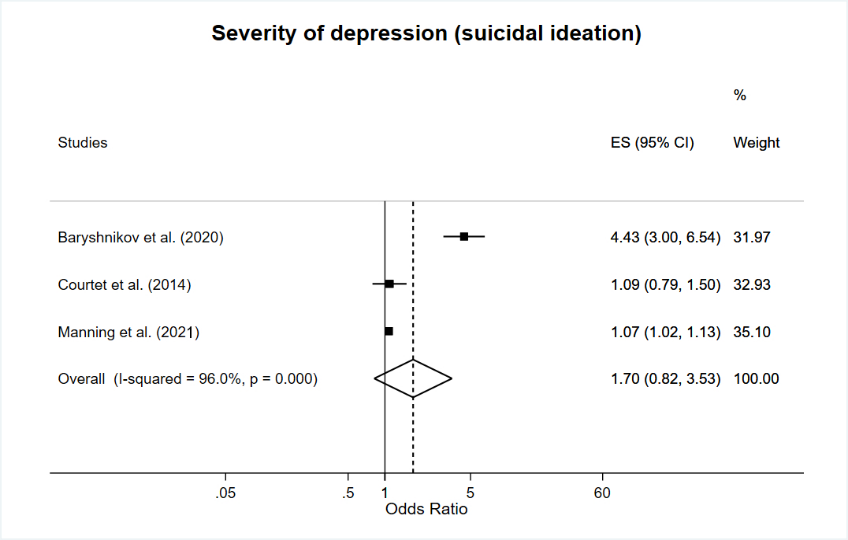


**Figure S1**. Forest plots for suicidal ideation predictors in adults with depression


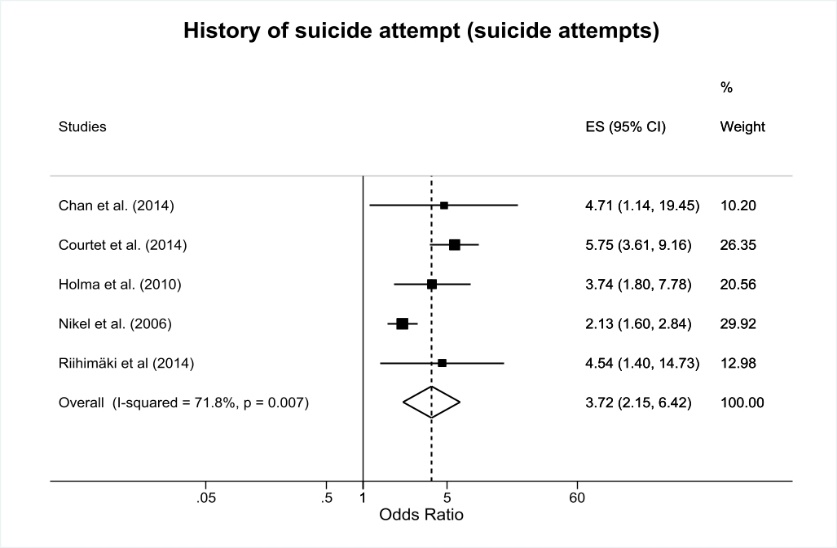

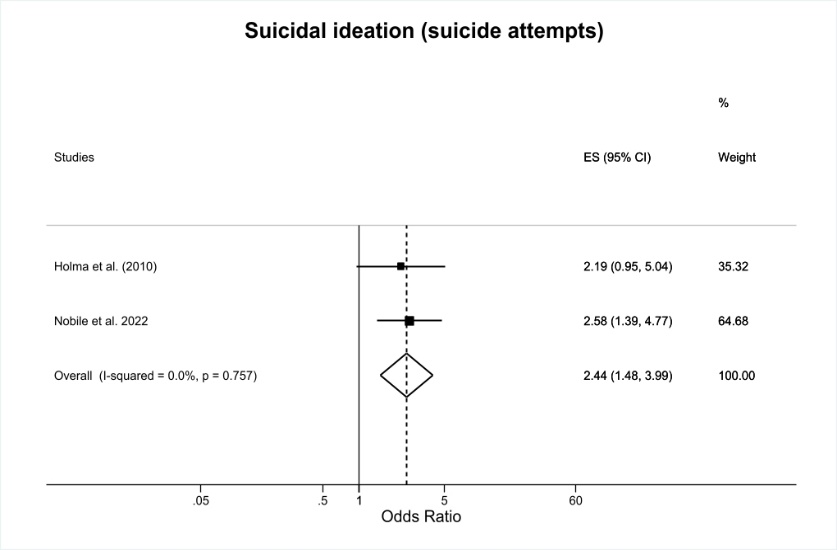

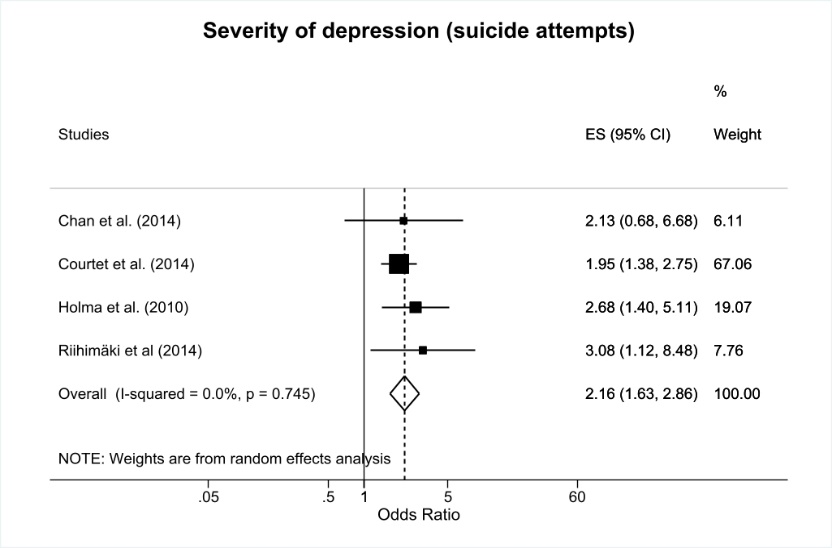

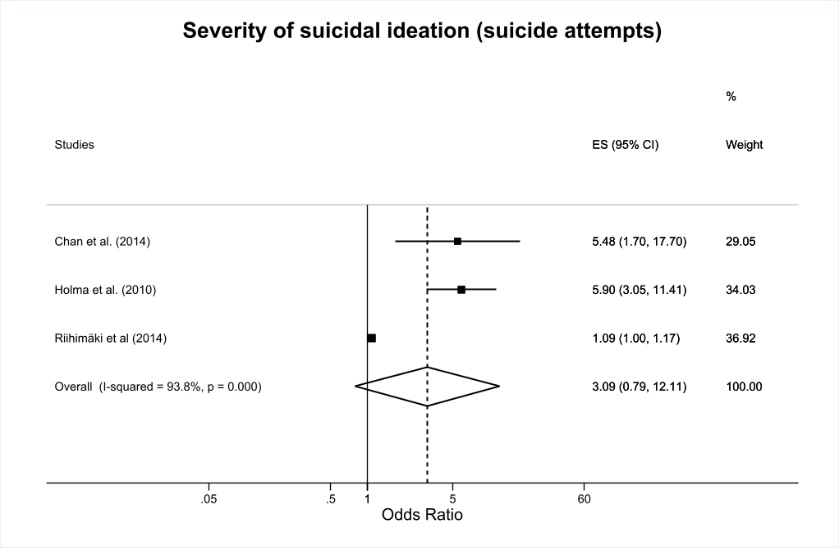


**Figure S2**. Forest plots for suicide attempts predictors in adults with depression


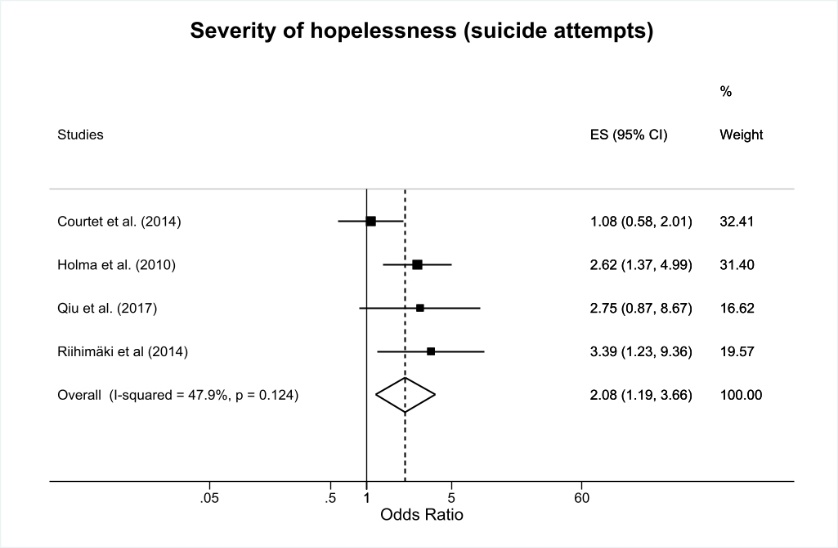

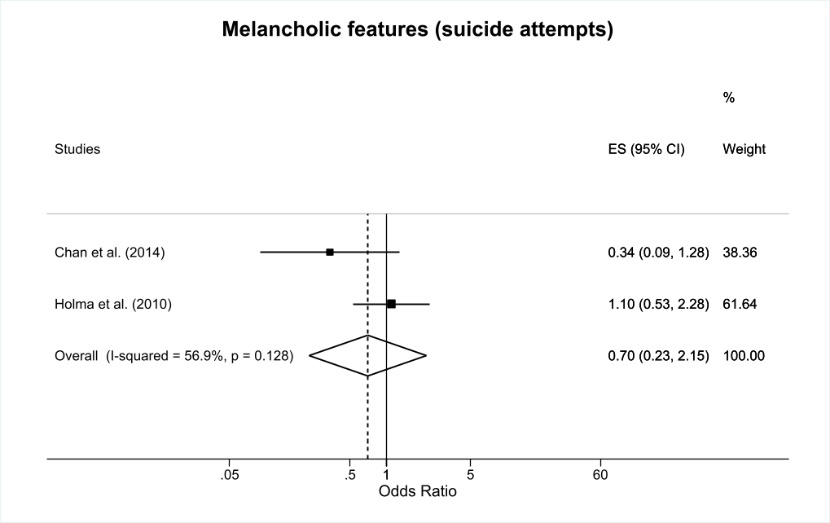

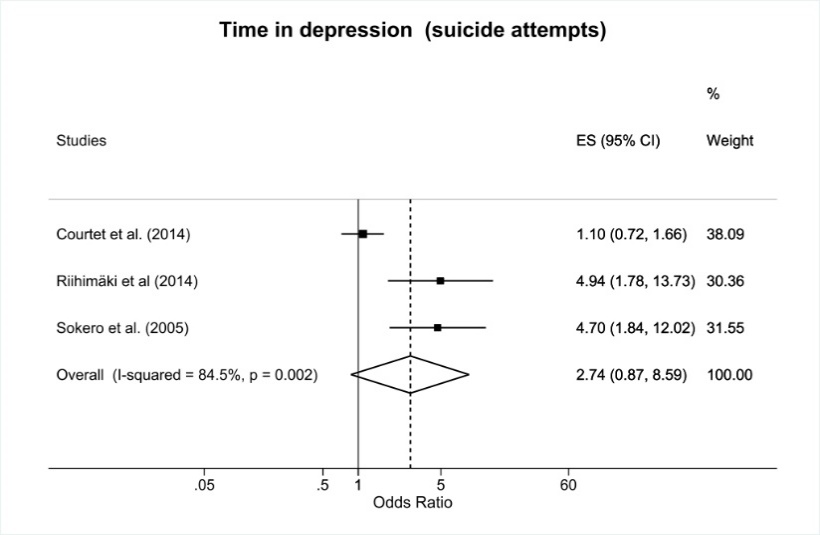

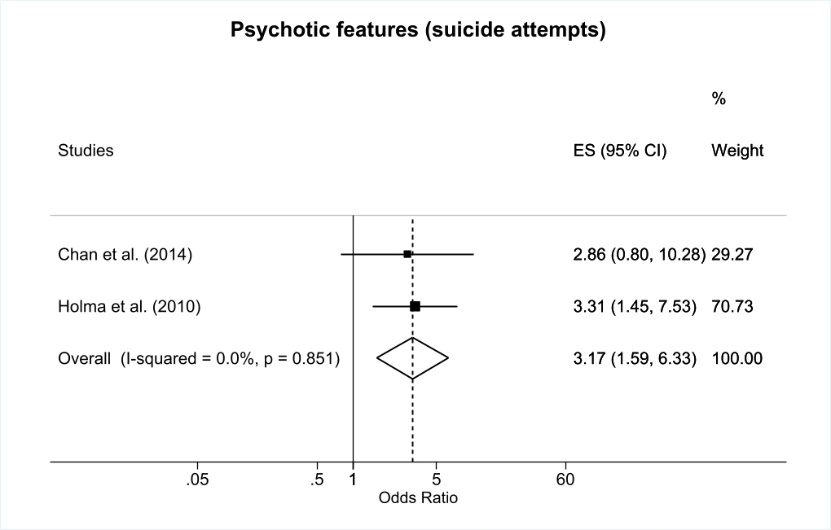


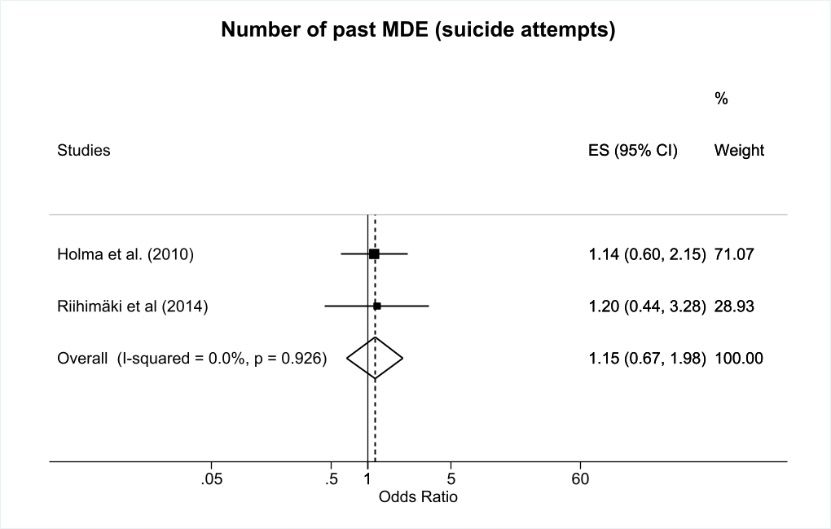

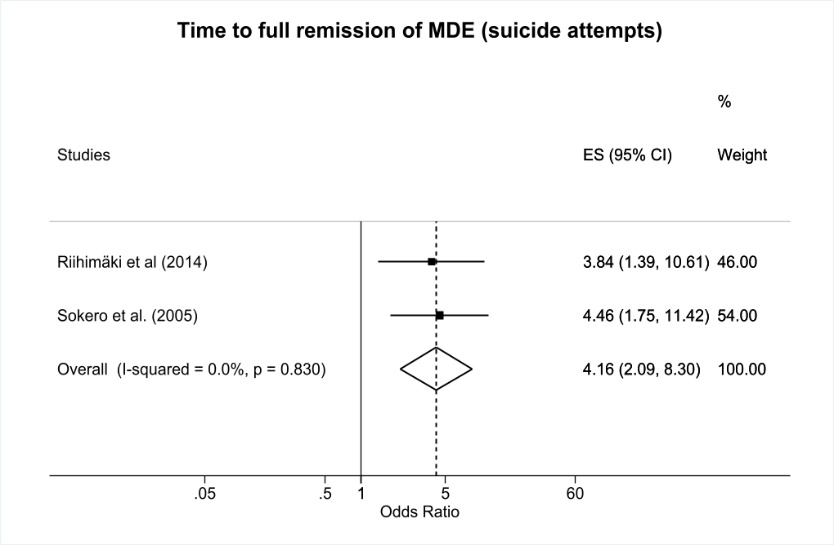

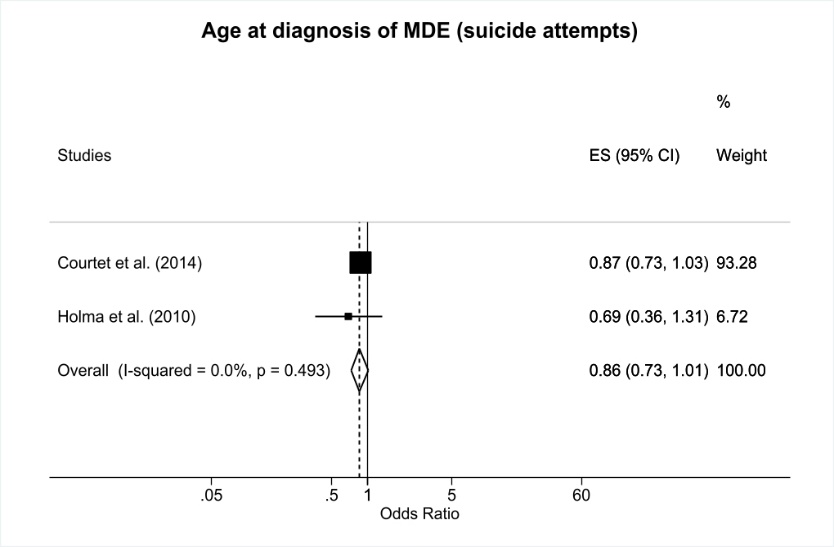


**Figure S2**. *(continued)*


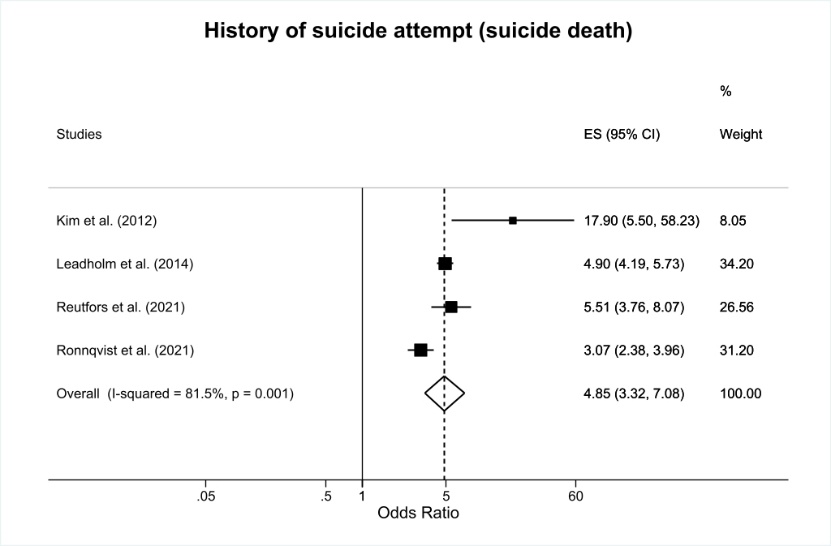

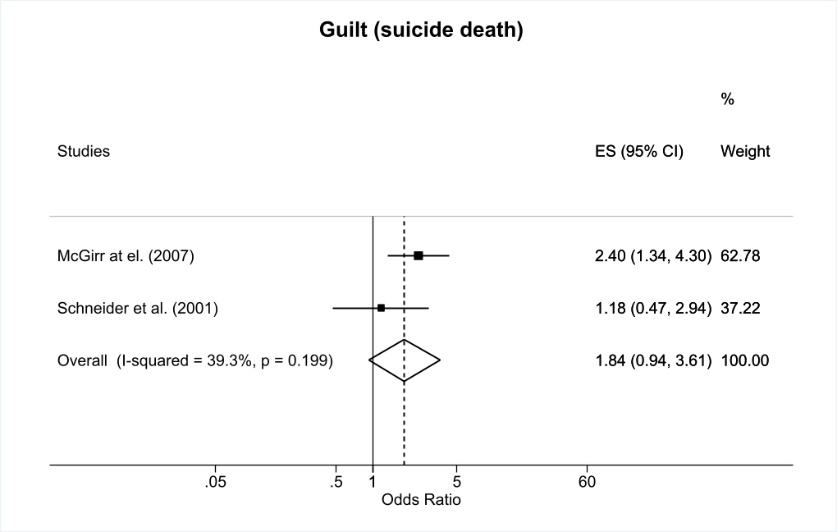

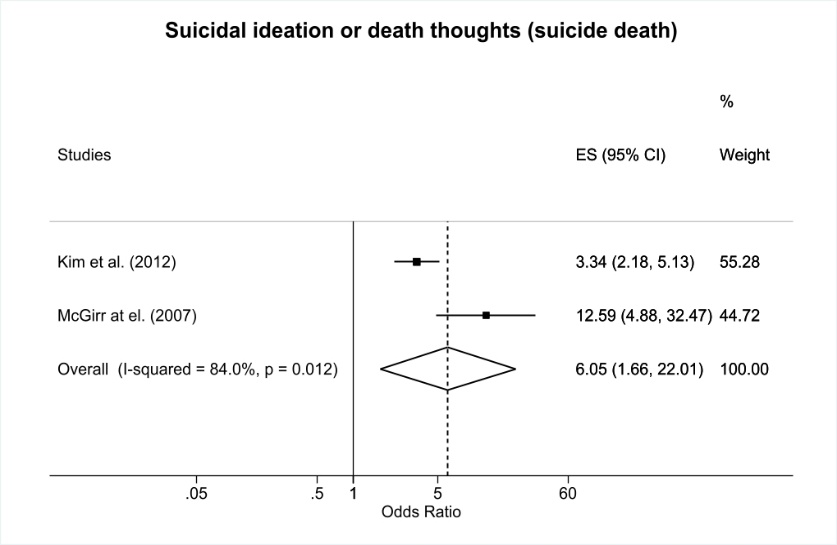

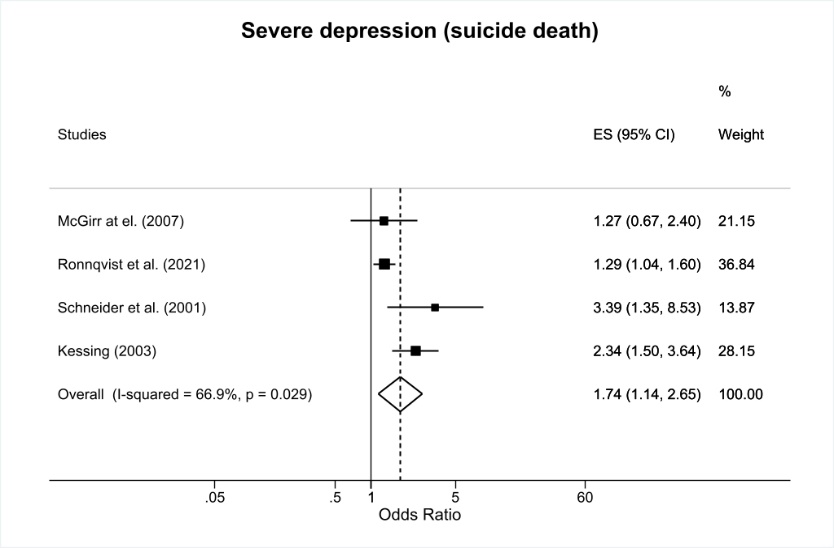

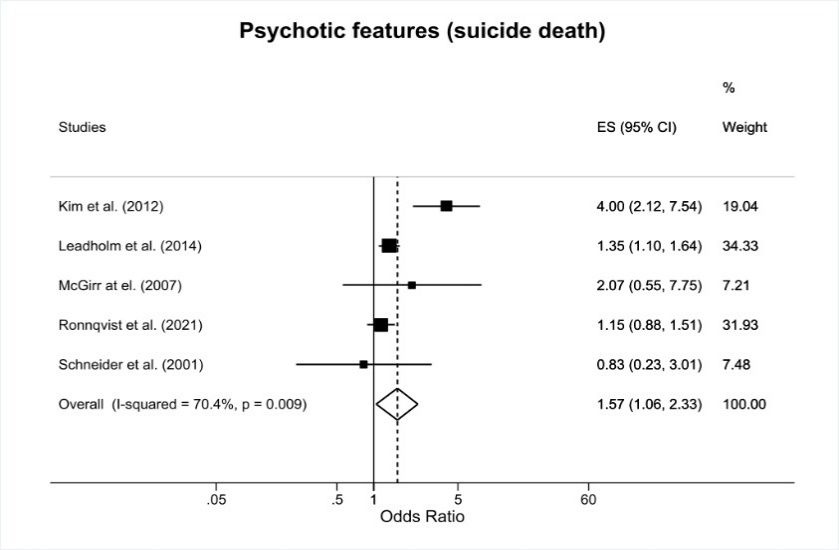

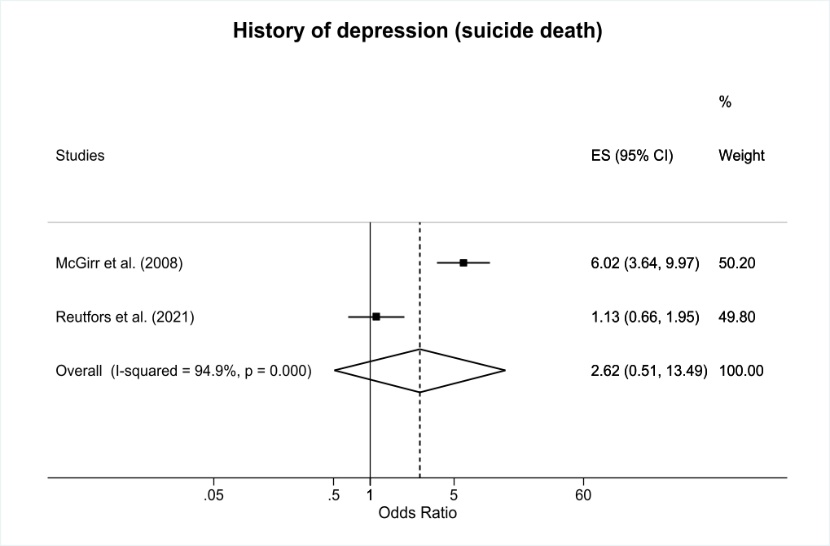

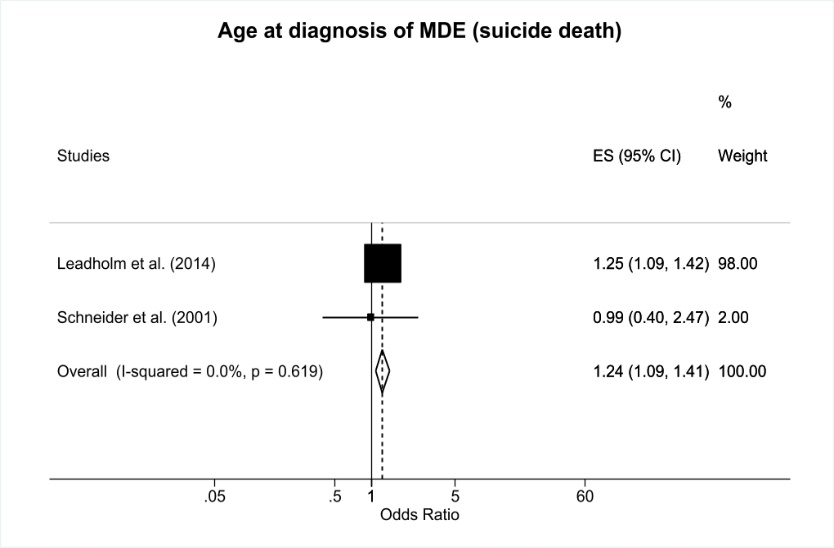

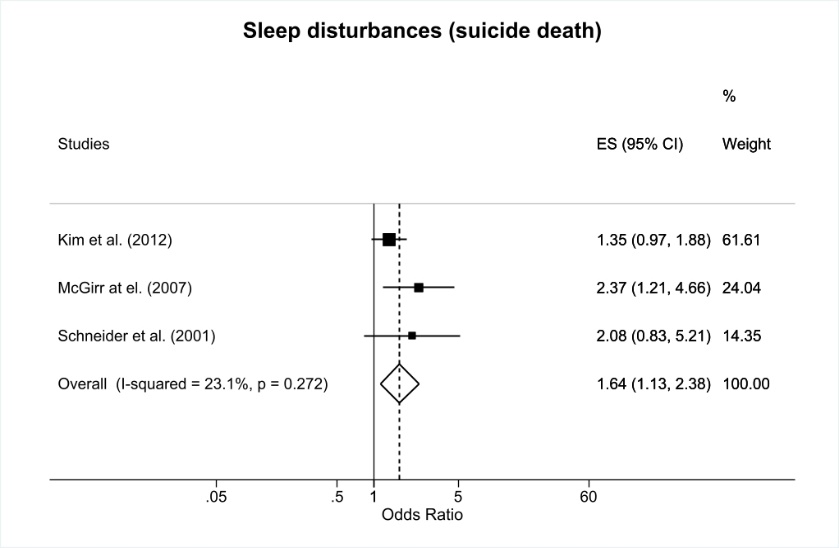


**Figure S3**. Forest plots for suicide death predictors in adults with depression
